# The R-enantiomer of ketorolac reduces ovarian cancer tumor burden in vivo

**DOI:** 10.1186/s12885-020-07716-1

**Published:** 2021-01-07

**Authors:** Martha M. Grimes, S. Ray Kenney, Dayna R. Dominguez, Kathryn J. Brayer, Yuna Guo, Angela Wandinger-Ness, Laurie G. Hudson

**Affiliations:** 1 Department of Pharmaceutical Sciences, College of Pharmacy, University of New Mexico, Albuquerque, New Mexico USA; 2 Division of Molecular Medicine, School of Medicine, University of New Mexico, Albuquerque, New Mexico USA; 3 Analytical and Translational Genomics Shared Resource, Comprehensive Cancer Center, University of New Mexico, Albuquerque, New Mexico USA; 4 Department of Internal Medicine, School of Medicine, University of New Mexico, Albuquerque, New Mexico USA; 5 Department of Pathology, School of Medicine, University of New Mexico, Albuquerque, New Mexico USA

**Keywords:** Ovarian cancer, Ketorolac, Rho-family GTPase, Rac1, Cdc42, Gene expression, RNA-seq

## Abstract

**Background:**

Rho-family GTPases, including Ras-related C3 botulinum toxin substrate 1 (Rac1) and cell division control protein 42 (Cdc42), are important modulators of cancer-relevant cell functions and are viewed as promising therapeutic targets. Based on high-throughput screening and cheminformatics we identified the R-enantiomer of an FDA-approved drug (ketorolac) as an inhibitor of Rac1 and Cdc42. The corresponding S-enantiomer is a non-steroidal anti-inflammatory drug (NSAID) with selective activity against cyclooxygenases. We reported previously that R-ketorolac, but not the S-enantiomer, inhibited Rac1 and Cdc42-dependent downstream signaling, growth factor stimulated actin cytoskeleton rearrangements, cell adhesion, migration and invasion in ovarian cancer cell lines and patient-derived tumor cells.

**Methods:**

In this study we treated mice with R-ketorolac and measured engraftment of tumor cells to the omentum, tumor burden, and target GTPase activity. In order to gain insights into the actions of R-ketorolac, we also performed global RNA-sequencing (RNA-seq) analysis on tumor samples.

**Results:**

Treatment of mice with R-ketorolac decreased omental engraftment of ovarian tumor cells at 18 h post tumor cell injection and tumor burden after 2 weeks of tumor growth. R-ketorolac treatment inhibited tumor Rac1 and Cdc42 activity with little impact on mRNA or protein expression of these GTPase targets. RNA-seq analysis revealed that R-ketorolac decreased expression of genes in the HIF-1 signaling pathway. R-ketorolac treatment also reduced expression of additional genes associated with poor prognosis in ovarian cancer.

**Conclusion:**

These findings suggest that R-ketorolac may represent a novel therapeutic approach for ovarian cancer based on its pharmacologic activity as a Rac1 and Cdc42 inhibitor. R-ketorolac modulates relevant pathways and genes associated with disease progression and worse outcome.

## Background

Ovarian cancer is the leading cause of death from gynecologic malignancies with a five-year patient survival of less than 50% [1]. The majority of women are diagnosed with advanced disease and recurrence after front line therapy is common [2, 3]. Unlike many other cancers, there are limited options for targeted therapeutics in ovarian cancer patients [3–6]. Therefore, there is a clinical need to identify additional strategies for effective treatment and management of ovarian cancer.

The Ras-homologous (Rho) family of small GTPases (Rac1, Cdc42, and Rho) are highly regulated signaling proteins that modulate downstream targets when bound to GTP and are inactive in the GDP bound state [7–11]. Based on the signaling outcomes from Rac1 or Cdc42 activation, overexpression of each protein has been implicated in cancer growth, progression and metastasis, chemoresistance, and for some tumors, poor patient outcomes [8, 11–22]. In ovarian cancer, we reported elevated Rac1 and Cdc42 protein levels in high grade vs. low grade tumors [23] and elevated mRNA expression of a constitutively active splice variant Rac1b in low grade ovarian tumors [23]. Rac1 overexpression in ovarian cancer is associated with early tumor recurrence [19, 24] and decreased patient survival [17, 19]. Based on experimental and clinical evidence, Rac1 and Cdc42 have been investigated as potential targets for development of cancer therapeutics [8, 10, 11, 14, 25, 26].

**Fig. 1:**
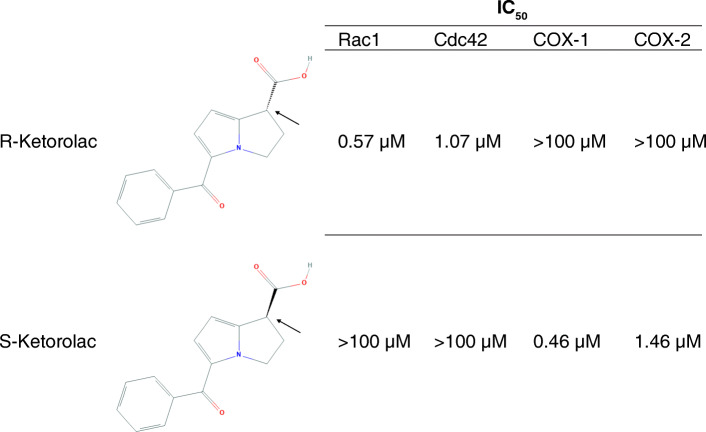
Distinct pharmacologic activities of the R- and S-enantiomers of ketorolac. Ketorolac is a chiral molecule administered as a 1:1 racemic mixture of S- and R-enantiomers, chemical structures are from PubChem. The arrow indicates the location of the chiral center. R-ketorolac inhibits Rac1 and Cdc42 with 50% inhibitory concentration (IC_50_) values of 0.57 and 1.07 μM for Rac1 and Cdc42, respectively as assessed in cell-based assays [23]. R-ketorolac displays negligible activity against cyclooxygenases (COX) 1 or 2 [26, 29]. S-ketorolac is considered the active component for ketorolac’s FDA-approved indication in pain management with selective activity against COX enzymes [29] and minimal activity against Rac1 and Cdc42 as measured in ovarian tumor cells [27]

Although selective inhibitors of Rho-family GTPases have been identified for preclinical testing, these agents have not been translated to the clinic. We conducted a high-throughput screen of the Prestwick library of off patent, FDA-approved drugs to identify activators and inhibitors of Rho GTPases [26]. The resultant findings coupled with cheminformatics approaches identified the R-enantiomers of a limited number of non-steroidal anti-inflammatory drugs (NSAIDs), R-naproxen and R-ketorolac, as inhibitors of Rac1 and Cdc42; these R-enantiomers lack the cyclooxygenase (COX) inhibitory activity of the S-enantiomers [26–28]. The S-enantiomers are the pharmacologic NSAIDs based on COX inhibition and lack activity against the Rac1 and Cdc42 GTPase targets [26]. R-ketorolac inhibits serum and epidermal growth factor-stimulated Rac1 and Cdc42 activation and downstream signaling at low micromolar concentrations [26, 27] (Fig. 1). Further testing found that R-ketorolac inhibited ovarian tumor cell adhesion, migration and invadopodia formation without cytotoxicity [23, 26, 27]. The inhibitory effects of R-ketorolac in cells are comparable to those of established Rac1 (NSC23766) and Cdc42 (CID2950007/ML141)-specific inhibitors [27, 30].

In this study, we tested the anti-tumor activities of R-ketorolac in a mouse ovarian cancer xenograft model of peritoneal disease. Treatment with R-ketorolac at dosing comparable to that achieved in patients receiving racemic (R−/S-) ketorolac inhibited tumor Rac1 and Cdc42 activity and decreased tumor burden. Based on RNA-sequencing (RNA-seq) analysis, R-ketorolac decreased tumor expression of genes associated with poor prognosis in ovarian cancer, some of which are represented in the HIF-1 signaling pathway. Taken together, these findings suggest that Rac1 may be a viable target for further drug development to attenuate aggressive tumor behaviors in ovarian cancer.

## Methods

### Cell culture and reagents

The human ovarian adenocarcinoma epithelial cell line SKOV3ip was obtained under a Material Transfer Agreement with MD Anderson Cancer Center (Houston, TX) [27]. These cells were then modified to express GFP by transfection with pEGFP-C1 (Catalog # 6084–1, Clontech) using Lipofectamine 2000 (Catalog # 11668030, Invitrogen Thermo Fisher Scientific) according to the manufacturer’s protocol. Positive cells were selected with G418 (Geneticin) at 0.3 mg/mL and further expanded for the establishment of stable cell lines. Both cell lines, SKOV3ip and SKOV3ip-GFP were authenticated using short tandem repeat analysis (performed by Promega). In vivo toxicity assessment following chronic R-ketorolac treatment was performed using SKOV3ip cells transduced with Red-shifted Luciferase using RediFect Red-FLuc-GFP lentiviral particles (Catalog # CLS960003, Perkin Elmer). Cells were then selected by flow sorting for GFP positive cells to generate SKOV3ip-RLuc-GFP.

SKOV3ip-GFP cells were cultured in RPMI-1640 media containing 5% FBS (Atlanta Biologicals), 2 mM L-glutamine, 1 mM sodium pyruvate, 25,000 U penicillin/streptomycin and 0.3 mg/mL G418. SKOV3ip-RLuc-GFP cells were cultured in RPMI-1640 media containing 10% FBS, 2 mM L-glutamine, 25,000 U penicillin/streptomycin. Cell culture media and reagents were purchased from Gibco (Life Technologies). R-ketorolac (cat# K235600) was purchased from Toronto Research Chemicals Inc., Toronto, Canada.

### Animal model

*Foxn1*^*nu*^ NU /J athymic nude female mice, aged 6–9 weeks, were purchased from The Jackson Laboratory (Bar Harbor, ME, stock number 002019). Female mice were selected because ovarian cancer is a gender specific disease. Mice were maintained at a controlled temperature of 22–23 °C, with a 12-h light/12-h dark cycle. Water and standard mouse chow were available ad libitum. All procedures were approved by the University of New Mexico Institutional Animal Care and Use Committee (IACUC) protocol (#18–200,772-HSC) and carried out in accordance with the NIH Guide for the Care and Use of Laboratory Animals. Mice appeared to be healthy, active, and of normal body weight for their age prior to any treatment or testing. Their health status was monitored throughout the experiment. No significant difference in body weight was observed with R-ketorolac treatment as has been previously reported [31]. No animals were taken off study due to morbidity or mortality. Mice were euthanized using a CO_2_ chamber in accordance with the recommendation of the Panel on Euthanasia of the American Veterinary Medical Association. Cervical dislocation was then carried out to confirm death before tumor analysis and tissue collection.

As an alternative to gavage for oral delivery, drug was administered in pills formed from transgenic dough (BioServ, Flemington, NJ, cat #S3472) [32]. This voluntary oral administration of drugs reduces stress on mice and has been shown to increase drug delivery into the bloodstream of rats [32, 33]. Briefly, R-Ketorolac was dissolved in 100% ethanol to a concentration of 5 mg/ml. Bromophenol blue was added to solutions at a final concentration of 0.1% as an aid to ensure even distribution of drug into the dough. Placebo pills were made using an equivalent volume of 100% ethanol containing 0.1% bromophenol blue. Dough was then pressed using 100 mg pill forms (Gallipot, St. Paul, MN). Pills were allowed to dry at room temperature overnight then removed from the forms and stored at 4 °C. Ketorolac, as racemic compound or individual enantiomer, was stable for at least 3 months in the pills as determined by high performance liquid chromatography (HPLC) (Additional File 2: Figure S1).

Mice were conditioned to placebo pills for two days prior to drug treatment. On day three, mice received placebo pills or pills containing 1 mg R-ketorolac per kilogram of body weight every 12 h. Consumption was confirmed by visual observation. For R-ketorolac short-term omental engraftment studies, SKOV3ip-GFP cells were pretreated with sterile saline or 10 μM R-ketorolac before intraperitoneal injection (i.p.) injection. Omenta were harvested from animals 18 h post-injection and placed in ice cold 1X phosphate-buffered saline (PBS) until imaged with an Olympus IX70 inverted fluorescent microscope and Olympus CellSens software. SKOV3ip-GFP cells were subsequently digested from the omentum using 10% NP-40 substitute (Sigma, cat# 74385) in 1X PBS for 30 min at 37 °C. Omenta were gently homogenized using disposable micropestles, centrifuged, and fluorescence intensity was measured using a Molecular Dynamics Spectramax M2 plate spectrophotometer (ex:480 nm, em:520 nm) [34].

For two-week tumor studies, 1 ×  10^6^ non-treated SKOV3ip-GFP cells were i.p. injected. All mice started R-ketorolac treatment one day prior to cell injection and continued treatment until conclusion of the study 14 days later. Mice were imaged using a Light Tools imaging system (Synopsys Optical Solutions, Westminster CO) with long pass GFP filters. Three images per mouse were captured to ensure all tumors were counted. Green fluorescent tumors were identified and counted in the peritoneal cavity as single tumors if there was a distinct border of non-fluorescent tissue. Tumor burden of the omentum occurred either as one large tumor or a bundle of tumors and was not included in the final analysis of tumor number. Total tumor counts from the peritoneal cavity were normalized to placebo treated animals within individual experiments. Blood was collected via cardiac puncture and ketorolac enantiomer concentrations were measured by HPLC analysis as described in Supplemental Methods (Additional File 1: Supplemental Methods). Tumor and tumor adjacent tissue was collected and stored in RNAlater (Qiagen, cat# 76104), RIPA cell lysis buffer (50 mM Tris-HCL pH 7.5, 150 mM sodium chloride (NaCl), 0.25% sodium deoxycholate, 10 mM sodium pyrophosphate, 10 mM β-glycerphosphate, 10 mM sodium fluoride (NaF), 1 mM EDTA. 1 mM PMSF (phenylmethylsulfonyl fluoride), 1 mM sodium orthovanadate (Na3VO4), 1 μg/mL pepstatin, 1 μg/mL leupeptin) or snap frozen using liquid nitrogen for further analysis.

For toxicity assessment studies at an increased R-ketorolac dose of 5 mg/kg/d, athymic nude female mice were purchased from Charles River Laboratory (Wilmington, MA, strain code 490). Mice were injected i.p. with 1 × 10^6^ SKOV3ip-RLuc-GFP cells suspended in sterile saline. Xenografts were established until tumors reached a bioluminescence imaging (BLI) value of ~ 3 × 10^8^ radiance (IVIS SpectrumCT, PerkinElmer, Waltham MA). Mice were conditioned to placebo pills once a day for no less than 3 days during xenograft growth, then randomized to receive placebo or 5 mg/kg/d R-ketorolac for 25 days. Blood was collected via cardiac puncture and 0.2–0.4 mL was added to Lithium Heparin tubes (BD Microtainer, Ref #365965). Samples were mixed thoroughly immediately after filling the tube by gently inverting the tube by hand. Samples were kept and analyzed at room temperature within 60 min of collection. For each sample analysis 100 μl of blood was added to Abaxis Comprehensive Diagnostic Profile cassette (Abaxis, Inc. Union City, CA Ref. # 500–0038-24) and values obtained using Abaxis VetScan2 analyzer.

### Flow cytometric GTPase effector binding assay

GTPase effector binding assays were carried out according to the protocols described previously [27, 35]. Frozen tumor samples were lysed without thawing with the addition of RIPA lysis buffer with the following modifications (no β-glycerphosphate,1 mM NaF and protease, 1% (v/v) NP-40 (nonyl phenoxypolyethoxylethanol) inhibitors consisting 10 μg/ml each of chymostatin, leupeptin, pepstatin and antipain). Protein concentrations were determined using GLISA quick protein concentration kit and lysates were adjusted to uniform protein concentrations of 0.5 mg/ml. Insoluble debris was removed by centrifugation at 14,000 RPM in a cold microfuge and the supernatants were incubated with GTPase effector coated beads (PAK1-PBD for Cdc42 and Rac1) for 1 h at 4 °C with rotation. Primary antibodies directed against Cdc42 or Rac1 and secondary antibody Alexa 488 were incubated with the beads for 1 h. Fluorescence intensity MCF (mean channel fluorescence) was used to measure the amount of active intracellular GTPase. MCF was measured by flow cytometry (Accuri C6, BD Biosciences). GTPase activity was calculated as (MCFsample group – MCFunstimulated negative control) / MCFstimulated positive control.

### RNA isolation and qRT-PCR

Samples of tumor tissue (10 mg) were snap frozen in liquid nitrogen and disrupted using an electric hand drill fitted with nuclease-free 1.5 mL pestles (Kimble-Chase, Vineland, NJ, cat#749521–1500). After disruption of tissue, buffer provided in the RNeasy Mini Kit (Qiagen, Valencia, CA, cat#74104) was added. The tissue lysate was homogenized using the QIAshredder (Qiagen, Valencia, CA, cat#79654) and RNA was isolated using the RNeasy Mini Kit according to the manufacturers’ protocols.

The integrity and quality of total RNA was evaluated by four different methods. The nucleic acid purity was conducted by measuring the UV absorbance using a NanoDrop ND 1000 UV-Vis Spectrophotometer (Thermo Fisher Scientific, Waltham, MA) to determine the concentration, 260/280, and 260/230 ratios. Only samples that met the following criteria were further tested, concentration ≥ 50 ng/μL, 260/280 of 2.0 ± 0.3, and 260/230 of 2.0 ± 0.4. RNA integrity was then assessed quantitatively by evaluating the intensity of the 28S and 18S ribosomal RNA (rRNA) bands from agarose gel electrophoresis. Samples that displayed distinct bands with no degradation were further analyzed as follows. RNA concentration was measured by fluorimetry using the Qubit 2.0 fluorometer (Invitrogen Thermo Fisher Scientific, Carlsbad, CA) with the Quant-it RNA Assay Kit (Invitrogen Thermo Fisher Scientific, Carlsbad, CA, cat#Q33140). The integrity and quality of total RNA was evaluated by using the Agilent 2100 Bioanalyzer (Agilent, Palo Alto, CA) with the RNA 6000 Nano Kit (Agilent, Palo Alto, CA, cat#5067–1511). Only RNA samples with a minimum RIN value ≥8.0 were used.

RNA was converted into cDNA using a High Capacity cDNA Reverse Transcription Kit (Applied Biosystems, Foster City, CA) and a TC-3000X Thermocycler (Techne Inc., Burlington, NJ). cDNA was generated from 500 ng of RNA of each sample. The resulting cDNA samples were diluted 1:4 with nuclease-free water.

Quantitative Real-Time polymerase chain reaction (qRT-PCR) for target analysis was conducted using six human primers: *CDC42*, *RHOA*, *RAC1*, *COX-1*, *COX-2*, and 18 s rRNA (Qiagen Quanti-Tect: QT01674442, QT00044723, QT00065856, qSTAR: HP204660, HP200900, and Qiagen Quanti-Tect: QT00199367, respectively). qRT-PCR for RNA-seq validation was conducted using eight human primers: *HMOX-1*, *CXCR4*, *VEGFA*, *KRT19*, *HK2*, *DUSP1*, *FAM42A*, and *IGFBP5* (Qiagen Quanti-Tect: QT00092645, QT00223188, QT01682072, QT00081137, QT00013209, QT00036638, QT00223258, and QT00047530, respectively). Fast SYBR® Green Master Mix (Applied Biosystems) was diluted with 2 μL of primer per reaction. Samples were loaded in quadruplicate in 96-well plates using 16 μL of master mix and 4 μL of sample per well. A nuclease-free water sample was used as a negative control, and 18 s rRNA was included as a positive control. Genes were amplified on a 7900 HT Fast Real-Time PCR System (Applied Biosystems) under the following conditions: 95 °C for 10 min, 40 cycles of (95 °C for 3 s, 60 °C for 30 s), and 95 °C for 15 s. Relative expression was calculated with the ΔΔct method, using 18 s rRNA for normalizing and analyzing the treated samples in reference to placebo samples.

### RNA sequencing analysis

Libraries were made using the Ion Total RNA-seq v2 kit (LifeTech, Carlsbad, CA) following the manufacturer’s suggested protocol, and sequenced on the Ion Proton S5 XL platform in the Analytical and Translational Genomics Shared Resource at the University of New Mexico Comprehensive Cancer Center. Using Kraken2, reads were first taxonomically classified [36, 37]. After classification, filterbyname.sh [38] was used to separate reads into *Homo sapiens* and *Mus musculus* fastq files. Reads that Kraken2 was unable to classify were placed in both species fastq files. Sequence reads were then mapped either to human genome hg38 or mouse genome mm10 using tmap (v5.10.11). Only reads mapping to exons were counted using HTseq (v0.11.1, [39]). Low expressing genes were excluded using a filtering threshold of 0.5 counts-per-millions (cpm) in at least three samples, and samples were normalized for library size. Differential gene expression was calculated using R packages edgeR and DESeq [39, 40], with a log2 fold-change threshold of 1.5 and an adjusted *p*-value of 0.5. The RNA-seq data is available for download from the NCBI BioProject database using study accession number PRJNA518157.

### Statistical analysis

Statistical analyses were performed using GraphPad Prism (GraphPad, San Diego USA) v8. Data was assessed to meet the corresponding statistical assumptions prior to statistical analysis. Statistical analysis was as follows: short-term omental engraftment was performed using unpaired t-test; long-term tumor burden, qPCR, and GTPase activity analyses was performed using One-way ANOVA and Dunnett’s multiple comparisons test. Animal hematology and biochemistry statistical analysis were performed using one-way ANOVA followed by Tukey’s multiple comparisons test. Regression on Order Statistics (ROS) procedure was performed to utilize blood chemistry data that was below the limit of detection (BLD). Analysis to generate adjusted means and standard deviations were run in R-Studio (Version 1.2.5019) using NADA Package [41]. For all analyses a *p*-value of less than 0.05 was considered significant.

## Results

### R-ketorolac decreases omental engraftment and tumor burden in vivo

**Fig. 2:**
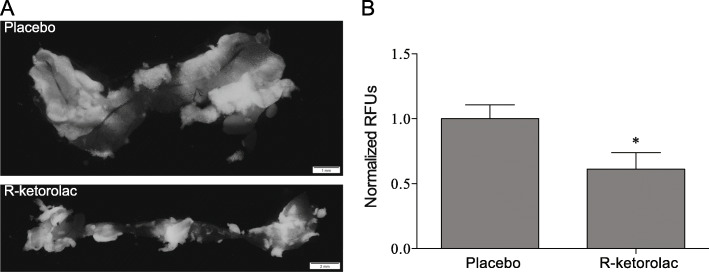
Oral administration of R-ketorolac reduces omental engraftment in vivo. Mice were injected i.p. with SKOV3ip-GFP cells and omental engraftment was assessed after 18 h as described in Methods. **a** Representative images of omenta isolated from animals receiving either placebo or R-ketorolac. **b** Omental engraftment was quantified by GFP fluorescence and normalized to placebo treated animals within individual experiments. These data represent the combined, normalized GFP fluorescence from three separate experiments with 12 total mice. There was a significant difference between placebo and R-ketorolac treated mice; * indicates p-value ≤0.05 using Student’s *t*-test

Ovarian cancer metastasis is largely confined to the peritoneal cavity and the omentum is a favored site for metastatic implantation and growth [17, 34, 42]. In mouse models, ovarian cancer cells rapidly home to the omentum after intraperitoneal injection [34, 43]. R-ketorolac treatment decreased the omental localization of SKOV3ip-GFP cells as measured by GFP fluorescence in a short term omental engraftment assay [34] (Fig. 2). These findings suggest that inhibition of Rac1 and Cdc42 GTPases by R-ketorolac reduces ovarian tumor cell adhesion to a metastatic site in vivo.

**Fig. 3:**
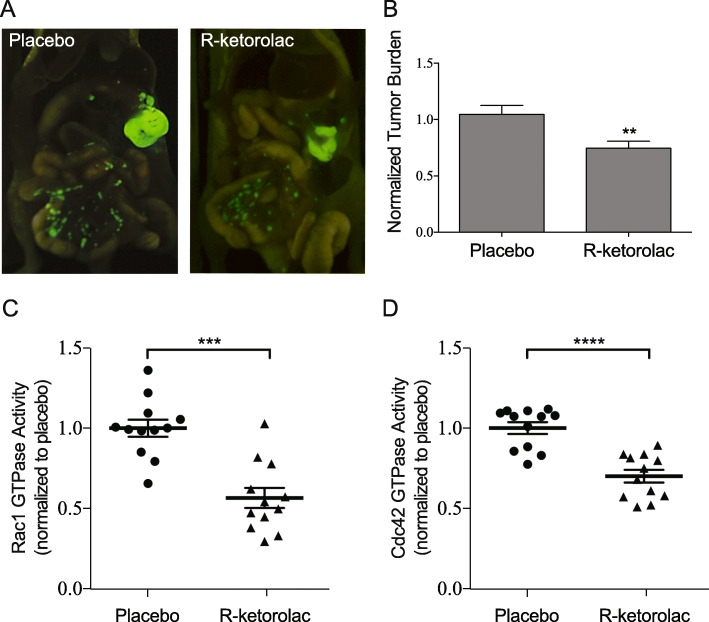
Oral administration of R-ketorolac reduces tumor burden in vivo and inhibits target GTPase activity in peritoneal tumors. Mice were injected i.p. with SKOV3ip-GFP cells and tumors were established for 14 days as described in Methods. **a** Representative images of the peritoneal cavity of mice treated with either placebo or R-ketorolac. **b** Tumor burden was quantified by counting visible tumor implants within the peritoneal cavity. Values are normalized to placebo control mice. Three images of each animal were captured to reveal tumors in each region of the peritoneal cavity. Data represents four separate experiments with 18 total mice. There is a significant difference between placebo and R-ketorolac treated mice; p-value ≤0.01 using Student’s *t*-test. GTPase activities of (**c**) Rac1 and (**d**) Cdc42 were measured in tumor lysates by a GTPase effector-binding assay as described in Methods. The data represent combined normalized activity from four separate animal experiments with GTPase activities measured in duplicate from three individual animals per experimental group, totaling 12 mice. The *p*-values were ≤ 0.001 and ≤ 0.0001 for Rac1 and Cdc42, respectively. Statistical analyses were performed using one-way ANOVA, followed by Dunnett’s multiple comparisons test. Vertical bars represent SEM

A two-week intraperitoneal tumor growth model was used to further test drug response. Mice received placebo or R-ketorolac at 1 mg/kg twice daily. The dose of R-ketorolac was selected to approximate the level of R-ketorolac administered to humans receiving the racemic drug [23, 28]. Mice injected with SKOV3ip-GFP ovarian tumor cells had significant peritoneal tumor implantation and growth after 14 days and tumor distribution in the animals was consistent with patterns of human disease dissemination (Fig. 3a). Mice treated with R-ketorolac had significantly fewer tumor implants compared to placebo treated animals (*p* ≤ 0.05) (Fig. 3a, b) and a trend of decreasing omental tumor weight (Additional File 3: Table S1).

### R-ketorolac treatment inhibits target GTPase activity in tumors

Oral administration of R-ketorolac for 2 weeks significantly decreased the activity of Rac1 and Cdc42 in tumor lysates when compared to those from placebo control groups (Fig. 3c, d). The magnitude of inhibition was similar to that observed in cells retrieved from the peritoneal cavity of ovarian cancer patients after racemic ketorolac administration [27]. R-ketorolac did not significantly decrease the mRNA expression of its pharmacologic targets *RAC1* or *CDC42* as measured by qPCR although a modest decrease was noted for *RHOA* (Additional File 4: Figure S2A). R-ketorolac does not inhibit the activity of RhoA [23], therefore the decrease in *RHOA* mRNA expression does not correspond to an effect on activity. Western blot analysis of tumor lysates shows that Rac1, Cdc42, or RhoA protein levels were not substantially decreased as a consequence of R-ketorolac treatment in vivo or after 5 days of treatment in cell culture (Additional File 4: Figure S2B, C). These findings suggest that the predominant effects of R-ketorolac in vivo are to inhibit the activity of Rac1 and Cdc42 as has been reported in vitro in biochemical and cell-based assays [26, 27].

### Toxicology of R-ketorolac

In order to evaluate potential drug toxicity, blood was collected via cardiac puncture from tumor bearing mice treated with a higher dose of R-ketorolac (5 mg/kg/d) and biochemical parameters were measured. There was no evidence for overt toxicity in mice treated with R-ketorolac for 25 days and few significant differences between blood samples from placebo or R-ketorolac treated mice (Table 1). Albumin decreased with R-ketorolac treatment compared to placebo resulting in values similar to non-tumor bearing control mice and near the reference range. Blood glucose in mice treated with R-ketorolac was increased when compared to placebo or control mice and modestly elevated above the reference range. Total bilirubin and alkaline phosphatase values were increased with R-ketorolac treatment compared to placebo, but these changes reflected a shift toward (alkaline phosphatase) or within (total bilirubin) the reference range. These findings suggest that extended treatment with R-ketorolac has minimal or no toxicity based on these common measures in blood samples.

**Table 1:** Toxicology Assessment of R-ketorolac. Hematology results are shown from mice bearing SKOV3ip-RLuc-GFP tumors and treated with placebo or R-ketorolac at 5 mg/kg/d as described in Methods. Control mice were non-tumor bearing and did not receive drug or placebo treatments and are diet, age, and living conditions matched to the other mice on study. Reference ranges are from a strain and age (6–8 weeks) matched Nu/Nu Mouse Biochemistry Technical Sheet from Charles River. Statistical analyses were performed using one-way ANOVA followed by Tukey’s multiple comparisons test. ^1^Data points missing due to reported hemolysis error; error may also result when hematocrit levels are high. ^2^Data averages and standard deviations calculated using Regression on Order Statistics (ROS) model to include points below limit of detection (BLD). *^, #^ Significant difference from placebo and control groups respectively. One, two, three, and four symbols indicates *p* ≤ 0.05, *p* ≤ 0.01, *p* ≤ 0.001, and *p* ≤ 0.0001, respectively)

	Placebo		BLD	R-Ketorolac		BLD	Control		BLD	Reference Range	
	Average ± SD	n=	n=	Average ± SD	n=	n=	Average ± SD	n=	n=	Average ± SD	n=
Albumin (g/dL)	4.43 ± 0.25	10		4.05 ± 0.40^*^	10		4.06 ± 0.09	5		2.8–4.0	113
Glucose (mg/dL)	222.3 ± 54.07	10		287.7 ± 81.31	10		214.20 ± 25.95	5		149–271	114
Total Bilirubin^1^ (mg/dL)	0.17 ± 0.06^##^	3		0.26 ± 0.05	9		0.32 ± 0.04	5		0.2–0.5	107
Alanine aminotransferase (U/L)	43.4 ± 19.36	10		32.30 ± 11.35	10		23.80 ± 4.55	5		31–115	105
Alkaline phosphatase^1,2^ (U/L)	16.58 ± 13.09^####^	9	3	28.97 ± 18.75^###^	10	2	68.6 ± 2.51	5		76–301	114
Amylase (U/L)	1027 ± 337	10		1069 ± 264	10		858 ± 90.13	5			
Blood Urea Nitrogen (mg/dL)	18.5 ± 2.92	10		16.4 ± 2.59	10		19.0 ± 3.61	5		11–39	114
Calcium (mg/dL)	11.53 ± 0.56	10		11.58 ± 0.79	10		10.76 ± 0.42	5		9.5–12.1	114
Creatinin^1,2^ (mg/dL)	0.16 ± 0.08	7	4	0.24 ± 0.13	9	3	0.20 ± 0.13	5	3	0.2–0.4	107
Globulin (g/dL)	1.5 ± 0.4	9		1.77 ± 0.43	10		1.36 ± 0.18	5			
Phosphorus (mg/dL)	9.59 ± 0.78	10		9.86 ± 1.90	10		6.65 ± 0.55	5		8.0–15.5	114
Sodium (mmol/L)	149 ± 2.98	10		151.3 ± 4.11^#^	10		145.0 ± 4.53	5		140.7–165.1	76
Total Protein (g/dL)	5.88 ± 0.36	10		5.79 ± 0.34	10		5.44 ± 0.13	5		4.8–6.6	114
Mouse Weight (g)	24.46 ± 1.42	10		23.67 ± 1.30	10		24.28 ± 2.13	5			

### RNA sequencing results of R-ketorolac treated tumor samples

To study the gene expression profiles of ovarian cancer xenografts following R-ketorolac treatment, we performed RNA-seq analysis using tumor tissue harvested from mice treated with R-ketorolac for two weeks or placebo control. On average, ~ 29 × 10^6^ reads were produced from each of the 6 libraries (range: 19.03–52.48 × 10^6^, Table 2). Prior to alignment, Kraken2 taxonomic sequence classification system was used to classify the RNA-seq reads as either human or mouse [36, 37, 44–46]. As would be expected, the majority of the reads in each sample mapped to the human genome (Table 2). On average 77% of the reads mapped to hg38 (range 66.5–82.9%; or 14.33–43.49 × 10^6^ reads, Table 2). However, an average of ~ 6 million reads per sample (~ 23%) mapped to the mouse genome (Table 2).

**Table 2:** RNA-Seq Statistics

Total Samples, n	6
Average Nucleotide Length	165 (155–171)
Average Total Reads, × 10^6^ (range)	29.5 (19.03–52.46)
Average Human Reads, × 10^6^ (range)	23.09 (14.33–43.49)
Average Mouse Reads, × 10^6^ (range)	6.77 (3.44–9.95)
Percentage of Human Reads (range)	77.6 (66.5–82.9)
Percentage of Mouse Reads (range)	23.4 (18.1–33.6)
Average Reads Mapped to hg38 Exons, × 10^6^ (range)	9.49 (5.38–18.77)
Average Reads Mapped to mm10 Exons, × 10^6^ (range)	2.81 (1.19–4.3)

**Fig. 4:**
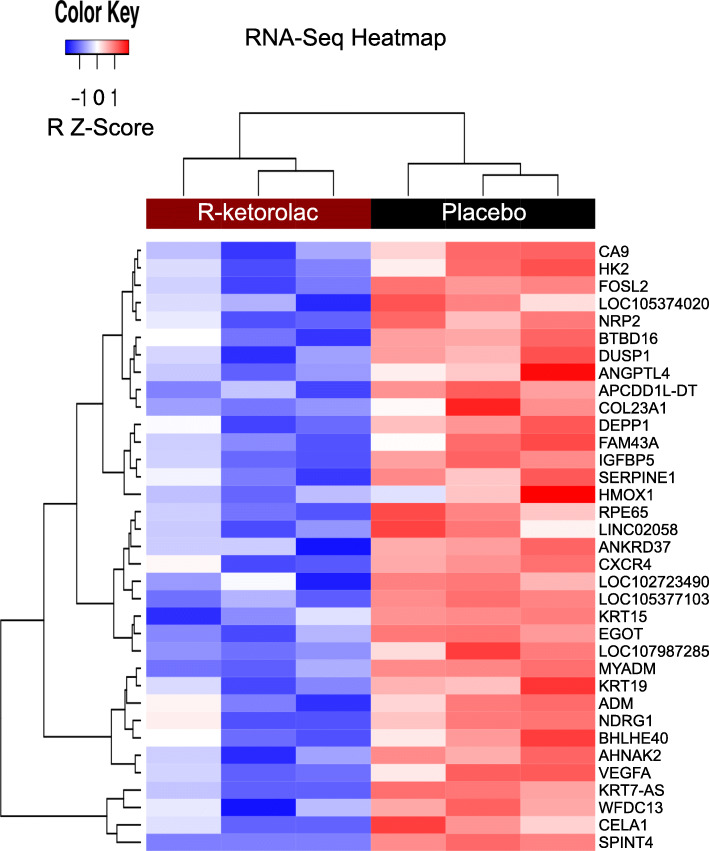
Heat map of 35 differentially expressed genes in tumors isolated from placebo versus R-ketorolac treated mice when aligned to the human genome. Differentially expressed genes are labeled on the right, samples are labeled on the top. Color key in the upper left indicates shading for up (red) and down (blue) regulated genes. Dendrograms at the top and side indicate relationship between the samples and differentially expressed genes, respectively. The color bar at the top indicates the sample conditions, R-ketorolac (dark red) and Placebo (black)

Only RNA-seq reads mapping to exons were counted for gene expression purposes, and on average ~ 40% of the reads in each genome landed on an exon (Table 2). Differential expression analysis was conducted on each species separately using gene-level expression data generated by summing exon-level counts. After filtering out genes with low expression, 18,176 human genes and 17,326 mouse genes remained for analysis. Using the human genome, thirty-five genes were identified as differentially expressed using a threshold of > 1.5-fold change and a false discovery adjusted *p*-value cut-off of 0.05. The gene expression signatures of the 35 down-regulated genes are summarized in Fig. 4. Additionally, when aligned to the mouse genome, 149 genes were identified as differentially expressed using a threshold of > 1.5-fold change and a false discovery adjusted p-value cut-off of 0.05. Within the gene expression signature of the 149 genes, 136 were down-regulated while 13 genes were up-regulated (Additional File 5: Figure S3).

**Fig. 5:**
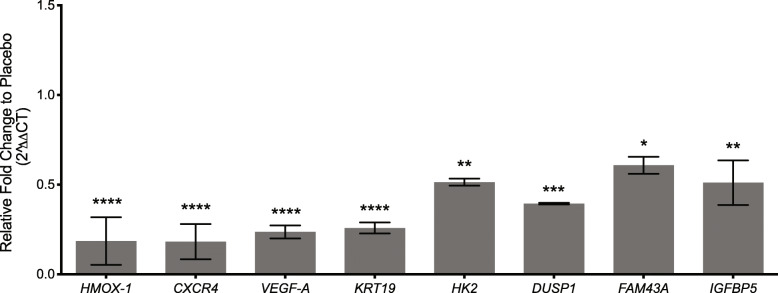
Expression of select genes to validate RNA-seq data from tumors of R-ketorolac treated mice relative to placebo. Gene expression levels from tumors obtained from placebo control and R-ketorolac treated mice were determined by qPCR as described in Methods. Values represent relative expression for R-ketorolac compared to placebo (1.0). *n* = 3. * indicates p-value ≤0.05, ** indicates p-value ≤0.01, *** indicates p-value ≤0.001, and **** indicates p-value ≤0.0001 using one-way ANOVA, followed by Dunnett’s multiple comparisons test

The significant gene list generated from the RNA-seq analysis aligned to the human genome included multiple genes associated with poor prognosis across cancers (*ADM*, *CXCR4*, *DUSP1*, *FAM43A*, *HK2*, *IGFBP5*, *NDRG1*, and *VEGFA*) [20, 47–58] and several genes (*CXCR4*, *HIF-1*, *VEGF*s, and *DUSP1*) have been associated with poor prognosis in epithelial ovarian cancer [17, 19, 22–24, 55, 59–67]. To verify the RNA-seq results, qRT-PCR was performed on 9 genes (*ADM*, *CXCR4*, *DUSP1*, *FAM43A*, *HK2*, *HMOX1*, *IGFBP5*, *KRT19*, and *VEGFA*) with the same RNA samples that were used for RNA-seq analysis. The findings confirm decreased expression in samples from R-ketorolac treated mice compared to placebo control (Fig. 5).

### R-ketorolac down-regulates genes within the HIF-1 signaling pathway

**Fig. 6:**
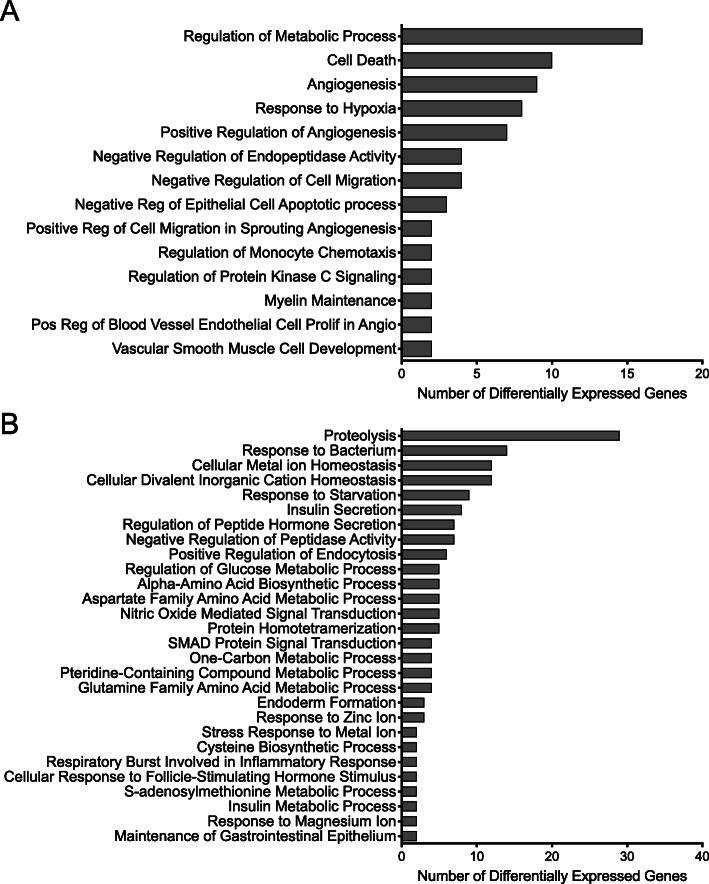
Bar plot of functional enrichment analysis of differentially expressed genes from RNA isolated from tumors of R-ketorolac treated mice. **a** The gene functional classification tool topGO produced 14 categories consisting of 20 genes when aligned to the human genome at adjusted p-value ≤0.05. **b** The gene functional classification tool topGO produced 28 categories consisting of 149 genes when aligned to the mouse genome at adjusted p-value ≤0.05. Columns show the number of genes related to each of the functional categories

Despite the relatively small number of differentially expressed genes that aligned to the human genome, enrichment analysis indicated that these genes affect several important biological functions. Using topGO to probe the Gene Ontologies database, we found the gene list to be enriched in 14 biological processes (Fig. 6a). Twenty genes (*RPE65, DEPP1, CELA1, ANGPTL4, MYADM, FOSL2, HK2, CXCR4, IGFBP5, WFDC13, SPINT4, HMOX1*, *DUSP1, VEGFA*, *SERPINE1*, *CA9, ADM*, *NRP2, COL23A1,* and *NDRG1*) in the first five topGO categories (Table 3) are associated with regulation of metabolic process, cell death, hypoxia, and angiogenesis (Table 3, Fig. 6a). The complete list of topGO categories and the differentially expressed genes that aligned to the human genome are reported in Additional File 6: Table S2. Analysis of the differentially expressed genes that aligned to the mouse genome using topGO to probe the Gene Ontologies database identified enrichment in 28 biological processes (Fig. 6b) encompassing 149 genes in the first five topGO categories associated with proteolysis, response to bacterium, cellular metal ion and divalent inorganic cation homeostasis, and response to starvation (Fig. 6b, Additional File 7: Table S3). *VEGFA* is the only gene present in the human and mouse genomes suggesting that R-ketorolac has distinct effects on tumor (human) or mouse (tumor microenvironment) tumor components.

**Table 3:** First 5 topGO Categories against Human Genome

Rank	Category	# genes	Genes
1	Regulation of metabolic process	16	*RPE65, DEPP1, CELA1, ANGPTL4, MYADM, FOSL2, HK2, CXCR4, IGFBP5, WFDC13, SPINT4, HMOX1, DUSP1, VEGFA, SERPINE1, CA9*
2	Cell death	10	*ADM, ANGPTL4, FOSL2, HK2, CXCR4, HMOX1, DUSP1, VEGFA, SERPINE1, NDRG1*
3	Angiogenesis	9	*ADM, CELA1, ANGPTL4, HK2, NRP2, HMOX1, COL23A1, VEGFA, SERPINE1*
4	Response to hypoxia	8	*ADM, ANGPTL4, HK2, CXCR4, HMOX1, VEGFA, NDRG1, CA9*
5	Positive regulation of angiogenesis	7	*ADM, CELA1, ANGPTL4, HK2, HMOX1, VEGFA, SERPINE1*

We further analyzed the differentially expressed significant human genes by using the kegga function in limma [68] to probe the Kyoto Encyclopedia of Genes and Genomes (KEGG) to determine if R-ketorolac had an effect on specific pathways. Against the human genome, the HIF-1 signaling pathway was significant (*p*-value 3.07E-05), with 4 of the differentially expressed genes present in this pathway (*HMOX1*, *VEGFA*, *SERPINE1*, *HK2*) (Table 4). For the human reads, the HIF-1 signaling pathway was also significant using GSEA, DAVID, AmiGO, and Reactome pathway analysis tools. For the mouse genome, the HIF-1 signaling pathway was also significant (p-value 0.005), with 5 of the differentially expressed genes present in this pathway (*NOS2, VEGFA*, *PDK1, EGF, SLC2A1*) (Additional File 8: Table S4). Although only *VEGFA* is shared between the mouse and human reads, R-ketorolac alters genes within the HIF-1 pathway in both the human and mouse components of the tumor (Additional File 9: Figure S4).

**Table 4:** Significant KEGG Pathways using Human Genome

Rank	Category	# genes	p-value	Genes
1	HIF-1 signaling pathway	4	3.07E-05	*HK2, HMOX1, VEGFA, SERPINE1*
2	Fluid shear stress and atherosclerosis	3	1.68E-03	*HMOX1, DUSP1, VEGFA*
3	Neomycin kanamycin and gentamicin biosynthesis	1	8.82E-03	*HK2*
4	*Staphylococcus aureus* infection	2	1.21E-02	*KRT15, KRT19*
5	AGE-RAGE signaling pathway in diabetic complications	2	1.30E-02	*VEGFA, SERPINE1*
6	Estrogen signaling pathway	2	2.40E-02	*KRT15, KRT19*
7	Nitrogen metabolism	1	2.97E-02	*CA9*

## Discussion

Despite evidence that elevated expression or activity of Rac1 is associated with worse prognosis in ovarian cancer [17, 19], there is limited knowledge on the impact of Rac1 or Cdc42 inhibition in vivo. We find that R-ketorolac, a dual Rac1/Cdc42 inhibitor, decreased tumor burden in both a short-term omental engraftment assay and a two-week tumor growth study. These findings are consistent with the observed effects of Rac1 silencing in a subcutaneous model of ovarian tumor growth [19] or treatment with zoledronic acid. Zoledronic acid inhibits GTPase prenylation and disrupts signaling of multiple small GTP binding proteins including Rac1 and Cdc42 [69–71]. Tumor weight and the number of tumor nodules of HEY8 ovarian cancer xenografts were decreased in response to zoledronic acid [69]. Taken together, these studies support potential benefits of inhibiting Rac1 and/or Cdc42 in ovarian cancer.

R-ketorolac is a component of the FDA-approved racemic drug administered for pain relief. The dose of R-ketorolac used in this study was selected to approximate human serum levels after administration of racemic drug [72]. The human equivalent dose of R-ketorolac partially inhibited Rac1 and Cdc42 and the magnitudes of inhibition were similar to those measured in samples from ovarian cancer patients who received the racemic drug [23]. Because the racemic drug is the form approved for use in patients, we conducted studies to compare tumor response following treatment with racemic ketorolac, R-ketorolac and S-ketorolac. Racemic drug and R-ketorolac, but not S-ketorolac, significantly decreased tumor burden in the two-week study (Additional File 10: Figure S5). Enantiomer-selective pharmacokinetics lead to greater retention of R-ketorolac in serum compared to S-ketorolac after administration of racemic drug in mice and humans [23, 28]. In addition, S-ketorolac is significantly converted to the R-enantiomer in mice but not humans [28]. As a consequence of ketorolac pharmacokinetics and S-enantiomer conversion [28], R-ketorolac levels were greater than S-ketorolac in all ketorolac treatment groups after two-week chronic treatment (Additional File 11: Figure S6) The studies reported in this manuscript reflect daily dosing of R-ketorolac in mice. In humans, multiple dosing regimens with racemic ketorolac over 5 days did not result in accumulation of either enantiomer [72]. The steady state blood concentrations of R-ketorolac at conclusion of the chronic administration (14 day) tumor study (~ 1.4 μg/ml) are lower than the maximal peak serum concentration (2.48 μg/ml) after single dose oral administration of R-ketorolac [28]. These data indicate that, as in human, R-ketorolac does not appear to accumulate with repeated dosing.

Inhibition of the target GTPases was evident in mice dosed with racemic ketorolac, which is consistent with the observed tumor response. Differences between R- and S-ketorolac were noted for the COX enzyme targets of S-ketorolac [28, 29, 73]. S-ketorolac treatment significantly decreased mRNA expression of *COX-1* and *COX-2* in tumor lysates from treated mice and both proteins were decreased in tumors from S- and racemic ketorolac, but not R-ketorolac, treated mice (Additional File 12: Figure S7). Decreased COX-1 protein expression after in vivo treatment with racemic ketorolac has been reported previously [74]. Collectively, these findings and published results [26–28] support the conclusion that R-ketorolac preferentially targets Rac1 and Cdc42 which could account for the observed anti-tumor action.

Despite partial inhibition of the GTPase targets at the human-equivalent dosing, RNA-seq analysis provided insights into the actions of R-ketorolac in vivo. The HIF-1 signaling pathway was identified as a significant KEGG pathway with key genes decreased in tumors from mice receiving R-ketorolac (Additional File 9: Figure S4). Interestingly, we detect increased expression of *HMOX-1, CXCR4, VEGFA and HIF1-α* in SKOV3ip-GFP cells overexpressing Rac1 protein by 2.78 fold (Additional File 14: Figure S9). Conversely, expression of these genes is decreased in tumors isolated from R-ketorolac treated mice as indicated in Figs. 4 and 5, Table 3 and Additional File 14: Figure S9C thereby illustrating reciprocal regulation of downstream response genes by increased or decreased Rac1 activity.

Hypoxia stimulates angiogenesis [75] and Rac1 signaling has been reported to promote angiogenesis [14] and Rac1 expression correlated with blood vessel invasion in a meta-analysis of multiple cancer studies [59]. Interestingly, R-ketorolac decreased expression of the angiogenic marker *VEGFA* (Additional File 14: Figure S9). High VEGFA is considered an indicator of poor prognosis in ovarian cancer [65, 66, 76]. Other genes identified by RNA-seq analysis as down-regulated by R-ketorolac are implicated in ovarian cancer. A meta-analysis indicated that high *CXCR4* expression was associated with poor prognosis in ovarian cancer [64] and *CXCR4* expression was significantly reduced in vivo after treatment with R-ketorolac. Eight genes that were associated with disease progression in an RNA-seq analysis of the metastatic microenvironment from high-grade serous ovarian cancer patient biopsies [53] were in the list of thirty-five genes down-regulated by R-ketorolac when compared to the human genome (*ADM*, *CXCR4*, *DUSP1*, *FAM43A*, *HK2*, *IGFBP5*, *NDRG1*, and *VEGFA*). R-ketorolac also modified gene expression of the host (mouse) component in the tumors. The significant KEGG pathways differed from those identified within the differentially expressed human genes with the exception of the HIF-1 signaling pathway. This observation suggests that R-ketorolac alters tumor-intrinsic gene expression and gene expression within the tumor microenvironment. Further studies will be required to delineate respective contributions of R-ketorolac on tumor versus tumor environment to the anti-cancer activity.

## Conclusions

We have shown that R-ketorolac has meaningful impact in an in vivo model of ovarian cancer. Inhibition of the GTPase targets led to reduced expression of pathways and genes associated with worse outcomes in ovarian and other cancers. We also reported benefit of R-ketorolac treatment in an aggressive genetic model of breast cancer [31]. It is interesting to note that retrospective studies find enhanced survival in breast cancer [77–79] and ovarian cancer patients [23] receiving ketorolac for post-operative pain relief. Clinical use of racemic ketorolac is restricted to five days because of toxicity largely attributed to COX inhibition by the S-enantiomer. We found that R-ketorolac was well tolerated over a twenty five day period with minimal impact on blood markers of renal or hepatic toxicity. R-ketorolac may hold promise for future clinical use in cancers with elevated Rac1 and/or Cdc42 expression or activity.

## Supplementary Information

**Additional file 1.** Supplemental Methods and Supplemental References.

**Additional file 2: Figure S1.** Enantiomer stability of ketorolac in the oral dosage form. Percent of each ketorolac enantiomer was determined by HPLC as described in Supplemental Methods (Additional File 1: Supplemental Methods). Analysis was conducted after pill storage at 4 °C for three months. Data presented are the average values from two pills.

**Additional file 3: Table S1.** Omental Weight.

**Additional file 4: Figure S2.** Expression of Rho-GTPases in tumors from placebo, R-ketorolac, S-ketorolac, and racemic ketorolac (R−/S-) treated mice. (A) Gene expression levels of *RAC1*, *CDC42* and *RHOA* were measured by qPCR as described in Methods. These data are combined from three separate experiments with a total of 12 mice. * indicates *p*-value ≤0.05 and ** indicates p-value ≤0.01 when compared to tumors of placebo mice and normalized to 18 s rRNA using one-way ANOVA, followed by Dunnett’s multiple comparisons test. (B) Western blot analysis of Rac1, Cdc42, and RhoA tumor protein levels as described in Supplemental Methods (Additional File 1: Supplemental Methods). GAPDH served as the loading control. Normalized values for R-ketorolac vs placebo (1.0) are Rac1 0.83, Cdc42 0.72 and RhoA 0.70 for bands detected by the respective mouse monoclonal antibodies. These are cropped images from the original western blots (Additional File 13: Figure S8). (C) To more specifically investigate the potential of R-ketorolac to modulate GTPase protein expression without potential interference of the mouse monoclonal antibodies reacting with mouse protein in the lysate, three independent cultures of SKOV3ip-GFP cells used for the xenografts were treated with 30 μM R-ketorolac for 5 days in culture. No significant differences in GTPase expression were detected as a consequence of R-ketorolac treatment.

**Additional file 5: Figure S3.** Heat map of 149 differentially expressed genes in tumors isolated from placebo versus R-ketorolac treated mice when aligned to mouse genome. Differentially expressed genes are labeled on the right and sample designations are labeled on the top. Color key in upper left indicates shading for up (red) and down (blue) regulated genes. Dendrograms at the top and side indicate relationship between the samples and differentially expressed genes, respectively. The color bar at the top indicates the sample conditions, R-ketorolac (red) and Placebo (black).

**Additional file 6: Table S2.** topGO Categories against the Human Genome.

**Additional file 7: Table S3.** topGO Categories against the Mouse Genome.

**Additional file 8: Table S4.** Significant KEGG Pathways using the Mouse Genome.

**Additional file 9: Figure S4.** HIF-1 Signaling Pathway as defined in the KEGG Pathway database. Highlighted are the differentially expressed genes found in the tumors isolated from R-ketorolac treated mice compared to placebo control when aligned to the human genome. Color key in upper right of figure indicates shading for up (red) and down (blue) regulated genes.

**Additional file 10: Figure S5.** Effect of racemic and S-ketorolac on tumor burden and GTPase activity in vivo. (A) Mice were injected i.p. with SKOV3ip-GFP cells and omental engraftment was assessed after 18 h as described in Methods. Representative images of omenta isolated from animals receiving either placebo or racemic ketorolac pills. (B) Omental engraftment was quantified by GFP fluorescence and normalized to placebo treated animals within individual experiments. These data represent the combined normalized GFP fluorescence from three separate experiments with 12 total mice. Oral administration of S-ketorolac and R−/S-ketorolac reduces tumor burden in vivo. (C) Mice were injected i.p. with GFP-expressing SKOV3ip and tumors were established for 14 days. Representative images are shown of mice treated with either placebo, S-ketorolac or R−/S-ketorolac. (D) Tumor burden was quantified by counting visible tumor implants within the peritoneal cavity and normalized to placebo control mice as described in the legend to Fig. 2. Data represents three separate experiments with S-ketorolac, *n* = 11; R−/S-ketorolac, *n* = 14. * indicates p-value ≤0.05 when compared to placebo control group. GTPase activity of (E) Rac1 and (F) Cdc42 were measured in tumor lysates by a GTPase effector-binding assay as described in Methods. The data represent combined normalized activity from four separate animal experiments with GTPase activities measured in duplicate from three individual animals per experimental group, *n* = 12 (n = 11 for S-ketorolac). GTPase activity for Rac1: p-value ≤0.0001 for S-ketorolac and for Cdc42 p-value ≤0.001 for R−/S-ketorolac; Cdc42 GTPase activity: p-value ≤0.001 for S-ketorolac and *p* ≤ 0.0001 for R−/S-ketorolac when compared to placebo group. Statistical analyses were performed using one-way ANOVA, followed by Dunnett’s multiple comparisons test. Vertical bars represent SEM.

**Additional file 11: Figure S6.** Ketorolac enantiomers in mouse serum after two-week drug treatment. Recovered ketorolac enantiomers in serum were analyzed by HPLC as described in Supplemental Methods (Additional File 1: Supplemental Methods). For each treatment group, grey bars represent average percent of R-enantiomer and black bars represent average percent of S-enantiomer. A predominance of R-ketorolac over S-ketorolac in all ketorolac treatment groups indicates an inter-conversion of S-ketorolac to R-ketorolac that occurs in mice as reported previously in the literature [28]. R-ketorolac represented approximately 88, 95, and 75% of the total recovered ketorolac from R−/S-ketorolac, R-ketorolac, and S-ketorolac treated animals, respectively after chronic administration for two weeks. Data represented are from three combined animal studies with a total of 12 mice.

**Additional file 12: Figure S7.** Expression of COX1 and COX2 in tumors from placebo, R-ketorolac, S-ketorolac, and racemic ketorolac (R−/S-) treated mice (all 1 mg/kg twice daily). (A) Gene expression levels of *COX1* and *COX2* were measured by qPCR as described in Methods. These data are combined from three separate experiments, *n* = 12. ** indicates p-value ≤0.01 and **** indicates p-value ≤0.0001 when compared to tumors of placebo mice and normalized to 18 s rRNA using one-way ANOVA, followed by Dunnett’s multiple comparisons test. (B) COX1 and COX2 protein levels were decreased with S-ketorolac or R−/S-ketorolac treatment, yet unaffected by R-ketorolac treatment. Tumor protein was isolated from placebo, R-ketorolac, S-ketorolac, or R−/S-ketorolac treated mice and analyzed by western blot analysis as described in Supplemental Methods (Additional File 1: Supplemental Methods). Representative blots from one of two independent experiments are shown. GAPDH served as the loading control.

**Additional file 13: Figure S8.** Raw images of western blot membranes used to detect Rho-GTPases in tumors from placebo and R-ketorolac treated mice. Membranes were cut between 25 and 37 kDa as guided using Precision Plus Protein Dual Color Standards (Bio-Rad Laboratories, Inc., cat# 1610374) before incubation with antibodies. This allowed probing of GAPDH (loading control) and the GTPase targets without a strip and re-probe procedure.

**Additional file 14: Figure S9.** Expression of select genes showing reciprocal regulation between Rac1 over-expressing cells (SKOV3ip-GFP-Rac1) and tumors from R-ketorolac treated mice. (A) Western blot verification of Rac1 over-expressed protein of SKOV3ip-GFP-Rac1 cells relative to SKOV3ip-GFP only cells. Total Rac1 protein was normalized to GAPDH loading control. This data is from two separate experiments and was compared using unpaired *t-*test. (B) Gene expression levels in SKOV3ip-GFP-Rac1 cells of *HMOX-1*, *CXCR4, VEGF-A* and *HIF1-α* were measured by qPCR as described in Methods. These data are combined from three separate experiments. They were compared to SKOV3ip-GFP and normalized to 18 s rRNA using unpaired two tailed *t*-test. (C) Gene expression levels of *CXCR4* and *HIF1-α* from tumors obtained from placebo control and R-ketorolac treated mice were determined by qPCR as described in Methods. Values are combined from three separate experiments and represent relative expression for R-ketorolac compared to placebo (1.0). Values were also normalized to 18 s rRNA and analyzed using unpaired two tailed *t*-test. For all panels * indicates p-value ≤0.05 ** indicates p-value ≤0.01 and **** indicates p-value ≤0.0001.

## Data Availability

The RNA-seq data generated is available for download from the NCBI BioProject database using study accession number PRJNA518157. All other dataset used and/or analyzed during the current study are available from the corresponding author on reasonable request.

## Abbreviations

ADM: Adrenomedullin
ANGPTL1: Angioprotein-like 1
CA9: Carbonic anhydrase 9
CDC42: Cell division cycle 42 gene
Cdc42: Cell division cycle protein
COX: Cyclooxygenase
CXCR4: Chemokine (C-X-C motif) receptor 4
DUSP1: Dual specificity phosphatase 1
EGLN3: Egl-9 family hypoxia inducible factor 3
FAM42A: Elongation factor-like GTPase 1
GAPDH: Glyceraldehyde-3-phosphate dehydrogenase
GLUT1: Glucose transporter 1
HIF-1: Hypoxia-inducible factor 1
HK2: Hexokinase 2
HMOX-1: Heme oxygenase 1
HSPA1B: Heat shock 70kD protein 1B
IGFBP5: Insulin-like growth factor binding protein 5
KRT19: Keratin 19
MCF: Mean channel fluorescence
MIOX: Myo-inositol oxygenase
MYADM: Myeloid-associated differentiation marker
NDRG1: N-myc downstream regulated 1
NRP2: Neuropilin 2
NSAID: Non-steroidal anti-inflammatory drugs
PAI-1: Serpine1
PHD: HIF prolyl hydroxylase
qRT-PCR: Quantitative Real-Time polymerase chain reaction
R−/S-ket: Racemic ketorolac
R-ket: R-ketorolac
RAC1: Ras-related C3 botulinum toxin substrate 1 gene
Rac1: Ras-related C3 botulinum toxin substrate 1 protein
RHO: Ras homolog gene
Rho: Ras homolog protein
RNA-seq: RNA-sequencing
S-ket: S-ketorolac
SD: Standard deviation
SEM: Standard error of the mean
SERPINE1: Serpin peptidase inhibitor, clade E, member 1
SLC2A1: Solute carrier family 2, member 1
VEGF: Vascular endothelial growth factor
