# Supplementary material for: The R-enantiomer of ketorolac reduces ovarian cancer tumor burden in vivo
Source: BMC Cancer. 2021 Jan 7;21:40. doi: 10.1186/s12885-020-07716-1 (PMC7791840; doi:10.1186/s12885-020-07716-1)
Supplement: Supplementary file 1 — Additional file 1. Supplemental Methods and Supplemental References. [file 12885_2020_7716_MOESM1_ESM.pdf]

SUPPLEMENTAL METHODS

Cell Culture and Reagents

SKOV3ip cells were modified to express GFP-Rac1 by transfection with pcDNA3-EGFP-Rac1-wt (Catalog # 12980, Addgene) as described for SKOV3ip-GFP cells under Methods. Rac1 overexpression quantification and verification was completed by western blot and qPCR. SKOV3ip-GFP and SKOV3ip-GFP-Rac1 cell populations were further enriched by flow sorting for GFP positive cells. SKOV3ip-GFP-Rac1 cell lines were maintained under the same selection as described for SKOV3ip-GFP cells (Methods).

In vivo Drug Dosing

Racemic (R-/S-) ketorolac tromethamine salt was purchased from Sigma (St. Louis MO, cat# K1136), S-ketorolac was purchased from Toronto Research Chemicals (Toronto Canada, cat# K235602) and dissolved in water or 100% ethanol, respectively. Mice received twice daily administration of placebo pills or pills containing racemic (R-/S-) ketorolac or S-ketorolac at 1 mg/kg of body weight.

Measurement of Omental Weight

Omental tissue was harvested and stored on ice in PBS for no more than 1 hour before analysis. Omenta were blotted dry on lab tissue and weight was measured using an analytical balance (Denver Instruments Company, A-160). There was a non-significant decreasing trend in omental weight for treated mice when compared to placebo mice (Additional File 3: Table S1).

High Performance Liquid Chromatography Separation of Ketorolac Enantiomers

Blood from mice was collected at sacrifice via cardiac puncture and stored at 4°C overnight. Samples were then centrifuged at 4000x*g* for 10 minutes at 4°C. Serum was separated from red cells and transferred into a fresh Eppendorf tube. Serum was then mixed 1:1 with 600 mM sulfuric acid and vortexed for 30 seconds at room temperature. Solution was vortexed with 3 ml diethyl ether and separated by centrifugation at 1000x*g* for 5 minutes at room temperature. The organic layer was removed and evaporated to dryness in a speedvac, then reconstituted at 37 °C in 200 µl of 0.1% formic acid in water. HPLC analysis was performed with a 50mm long 5µm silica guard column (Phenomenex, 03B-4053-N0) attached to a Partisil® 5 µm ODS (3) 85 Å LC Column 150 x 4.6 mm (Phenomenex, 00F-0120-E0) followed by a reverse phase Lux 5 µm Cellulose-3 50 x 4.6 mm column (Phenomenex, 00G-4493-E0). The columns were equilibrated with acetonitrile/0.1% Formic acid in water (25:75) at a flow rate of 2 ml/min. A standard curve of racemic ketorolac in water was generated based on an injection volume of 10 µl. Ketorolac was detected using a UV spectrometer set at UV310. Retention times for R-ketorolac and S-ketorolac were 5.0 minutes and 5.9 minutes respectively, and were validated against each individual enantiomer (1).

RNA Isolation and qRT-PCR from *in vivo* Tumor Samples and Cells in Culture

RNA was isolated from sample tumors, converted into cDNA, and target analysis was performed by qRT-PCR as previously described in Methods, using the following primers: *COX-1*, *COX-2*, *CXCR4*, *Hif1-α*, and 18s rRNA (qSTAR: HP204660, HP200900, Qiagen Quanti-Tect: QT00223188, QT00083664, and QT00199367, respectively).

Plated cells were washed 2x with PBS and lysed with buffer provided in the RNeasy Mini Kit (Qiagen, Valencia, CA, cat#74104). Cells were lifted from the plate with a cell scraper and cell lysate was homogenized using the QIAshredder (Qiagen, Valencia, CA, cat#79654). RNA was isolated using the RNeasy Mini Kit according to the manufacturers' protocols. RNA was converted into cDNA and target analysis was performed by qRT-PCR as described in Methods, using the following primers: *HMOX-1*, *CXCR4*, *VEGFA*, *Hif1-α*, and 18s rRNA (Qiagen Quanti-Tect: QT00092645, QT00223188, QT01682072, QT00083664, and QT00199367, respectively).

Protein Isolation from *in vivo* Tumor Samples and Cells in Culture

Conditions for tumor protein isolation were modified from protocols provided in Zakharchenko et al., 2011 (2) and Axxora An Enzo Company website as described previously (3). Frozen tissue was weighed (10-20 mg) and 250 µL of RIPA buffer, as described in Methods, was added with a protease and phosphatase inhibitor cocktail (Thermo Scientific #78445). Tissue was then homogenized using an electric drill with a plastic pellet pestle (Kimble Chase #7495150000) attached. Homogenized lysates were incubated on ice for 30 minutes, vortexed, and briefly sonicated. Lysates were incubated for 30 additional minutes and centrifuged at 12,000 rpm for 20 minutes at 4°C. The supernatant was stored in multiple aliquots at -80°C.

Plated cells were collected by removing media and rinsing twice with PBS. As described for tumor lysates above, the cells were then lysed with RIPA buffer plus protease and phosphatase inhibitor cocktail. Cell lysate was then incubated on ice for 10 minutes, vortexed, and briefly sonicated. Cell lysate was then centrifuged at 14,000 rpm for 10 minutes at 4°C. The supernatant was stored in multiple aliquots at -80°C. Proteins extracted from frozen tumor tissues and cultured cell lysate were quantified using a Pierce BCA protein assay kit (Thermo Scientific #23227).

Western Blot Analysis

For the western blots, 10 µg of tumor lysate protein from 3 mice was pooled totaling 30 µg/lane. Equal amounts of cell lysate at 20 µg/lane was used. All proteins were resolved by SDS-PAGE, transferred to nitrocellulose membranes, blocked with 5% non-fat milk for 1 hour at 4°C, and then blotted with the following antibodies and conditions. Antibodies directed against targets were from the reputable sources, with evidence of validation of specificity. Antibodies were used according to manufacturers' specifications and carefully assessed in our laboratory to ensure that individual proteins of the anticipated molecular weight were identified. Antibodies are as follows; COX-1 (Cell Signaling, Inc. #4841); COX-2 (Cell Signaling, Inc. D5H5 #12282); anti-Rac1 mouse mAb (CytoSkeleton, #ARC03 and BD Transduction #610650); anti-Cdc42 mouse mAb (CytoSkeleton, #ACD03 and BD Transduction #610928); and anti-RhoA (CytoSkeleton, #ARH04). Secondary antibodies anti-Mouse IgG HRP-linked (Cell Signaling, Madison, WI cat. #7076) were used at 1:1,000 dilution in 1% milk in TBST (13 mmol/L Tris-HCL, 150 mmol/L NaCl. 0.05% Tween-20 at pH 7.4). Blots were stripped at 50°C in stripping buffer (62.5 mmol/L Tris-HCL pH 6.7, 100 mmol/L β-mercaptoethanol, 2% SDS) for 30-60 minutes, followed by thorough rinsing with MilliQ water and three 10-minute washes in TBST. The blots were then reprobed with GAPDH mouse Mab MAB374 (Millipore Sigma, Burlington, MA) at 1:1000 dilution in 5% bovine serum albumin in TBST. Western blot images were acquired on ProteinSimple FluorChem R system and analyzed using AlphaView SA software (ProteinSimple, San Jose, CA).

References

1. Vakily M, Corrigan B, Jamali F. The Problem of Racemization in the Stereospecific Assay and Pharmacokinetic Evaluation of Ketorolac in Human and Rats. Pharm Res [Internet]. 1995 Nov 1 [cited 2019 May 21];12(11):1652–7. Available from: https://doi.org/10.1023/A:1016245101389

2. Zakharchenko O, Greenwood C, Alldridge L, Souchelnytskyi S. Optimized Protocol for Protein Extraction from the Breast Tissue that is Compatible with Two-Dimensional Gel Electrophoresis. Breast Cancer Basic Clin Res [Internet]. 2011 Mar 10 [cited 2019 Jan 17];5:37–42. Available from: https://www.ncbi.nlm.nih.gov/pmc/articles/PMC3076014/

3. Peretti AS, Dominguez D, Grimes MM, Hathaway HJ, Prossnitz ER, Rivera MR, et al. The R-Enantiomer of Ketorolac Delays Mammary Tumor Development in Mouse Mammary Tumor Virus-Polyoma Middle T Antigen (MMTV-PyMT) Mice. Am J Pathol [Internet]. 2018 Feb 1 [cited 2018 Feb 12];188(2):515–24. Available from: http://ajp.amjpathol.org/article/S0002-9440(17)30445-5/abstract
