# Supplementary figures and images for: The R-enantiomer of ketorolac reduces ovarian cancer tumor burden in vivo
Source: BMC Cancer. 2021 Jan 7;21:40. doi: 10.1186/s12885-020-07716-1 (PMC7791840; doi:10.1186/s12885-020-07716-1)

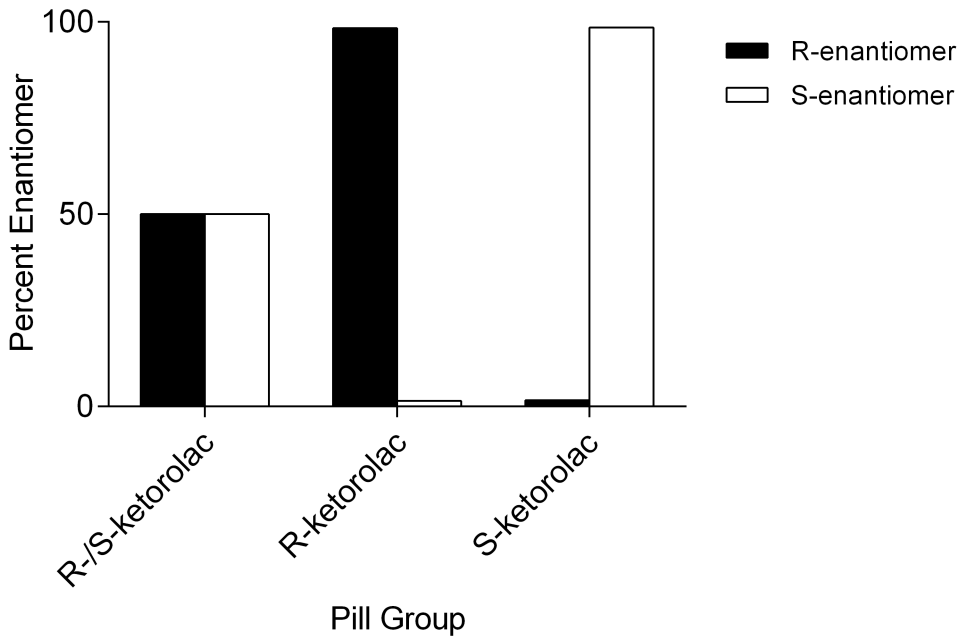

Supplement: Supplementary file 2 — Additional file 2: Figure S1. Enantiomer stability of ketorolac in the oral dosage form. Percent of each ketorolac enantiomer was determined by HPLC as described in Supplemental Methods (Additional File 1: Supplemental Methods). Analysis was conducted after pill storage at 4 °C for three months. Data presented are the average values from two pills. [file 12885_2020_7716_MOESM2_ESM.pdf]

A

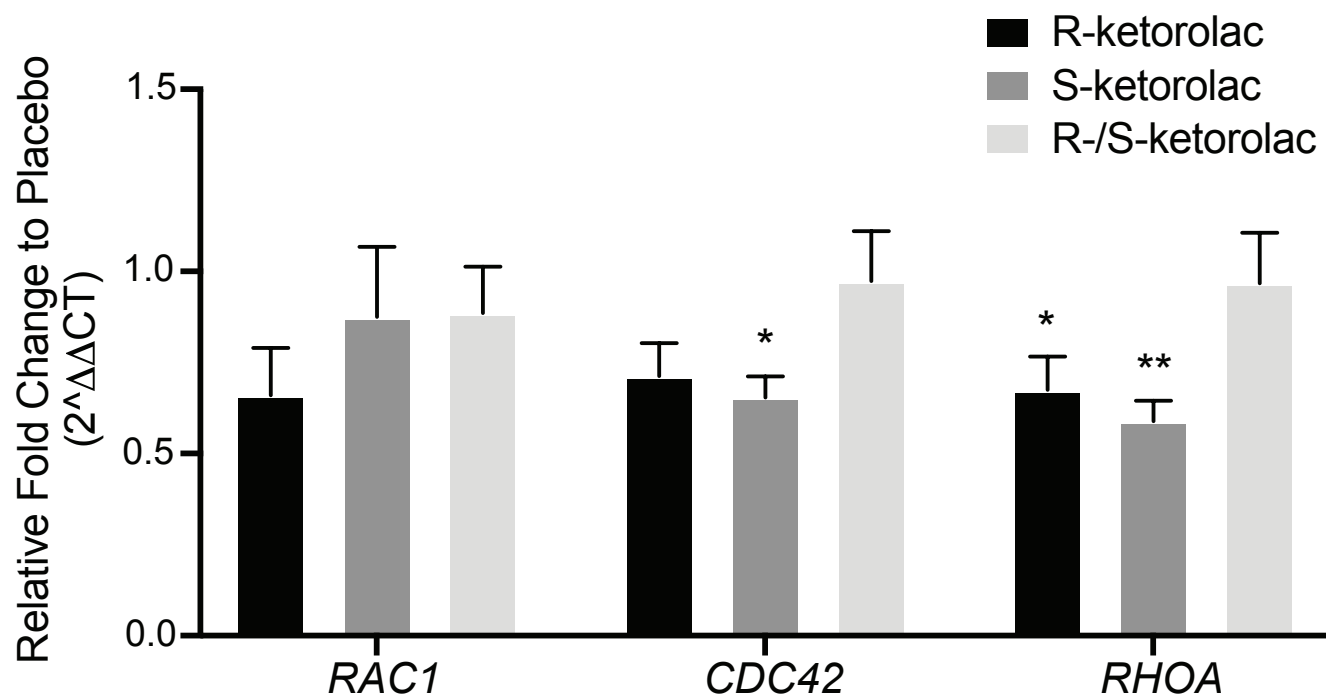

B

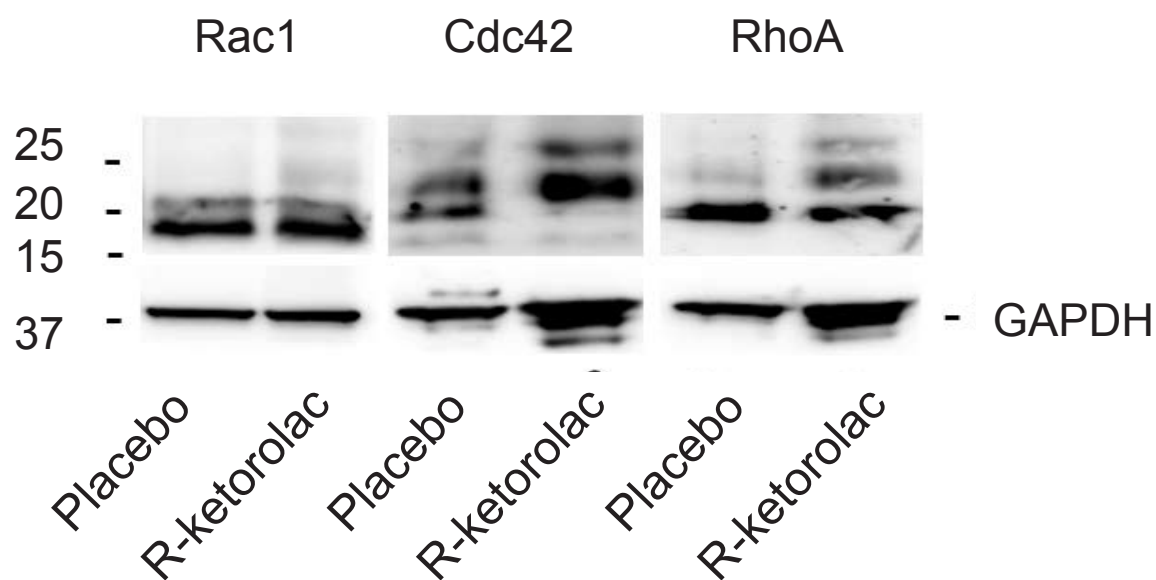

C

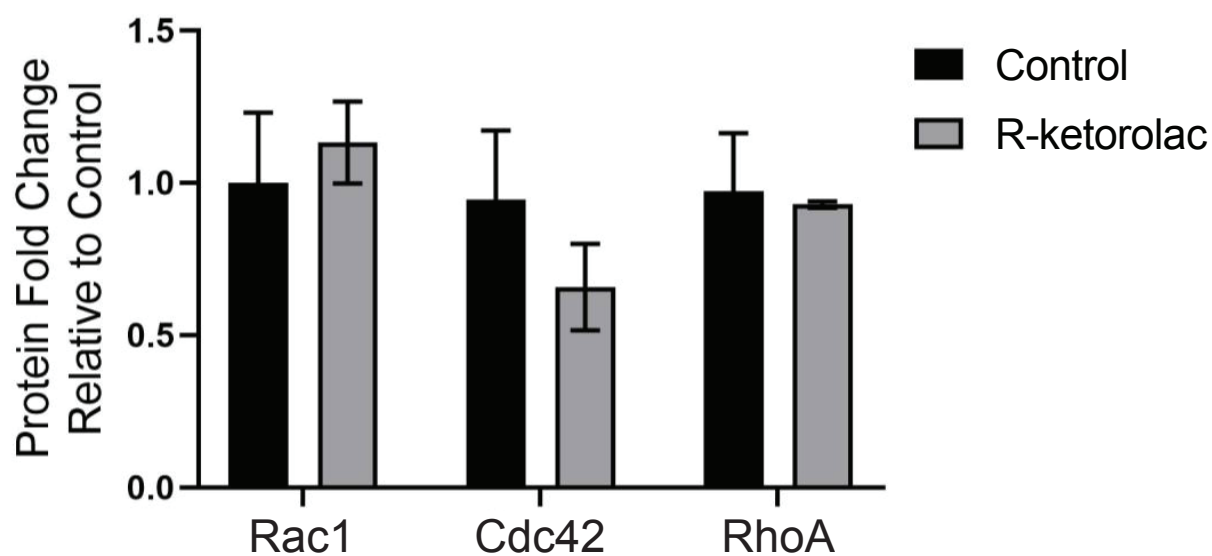

Supplement: Supplementary file 4 — Additional file 4: Figure S2. Expression of Rho-GTPases in tumors from placebo, R-ketorolac, S-ketorolac, and racemic ketorolac (R−/S-) treated mice. (A) Gene expression levels of RAC1, CDC42 and RHOA were measured by qPCR as described in Methods. These data are combined from three separate experiments with a total of 12 mice. * indicates p-value ≤0.05 and ** indicates p-value ≤0.01 when compared to tumors of placebo mice and normalized to 18 s rRNA using one-way ANOVA, followed by Dunnett’s multiple comparisons test. (B) Western blot analysis of Rac1, Cdc42, and RhoA tumor protein levels as described in Supplemental Methods (Additional File 1: Supplemental Methods). GAPDH served as the loading control. Normalized values for R-ketorolac vs placebo (1.0) are Rac1 0.83, Cdc42 0.72 and RhoA 0.70 for bands detected by the respective mouse monoclonal antibodies. These are cropped images from the original western blots (Additional File 13: Figure S8). (C) To more specifically investigate the potential of R-ketorolac to modulate GTPase protein expression without potential interference of the mouse monoclonal antibodies reacting with mouse protein in the lysate, three independent cultures of SKOV3ip-GFP cells used for the xenografts were treated with 30 μM R-ketorolac for 5 days in culture. No significant differences in GTPase expression were detected as a consequence of R-ketorolac treatment. [file 12885_2020_7716_MOESM4_ESM.pdf]

Color Key

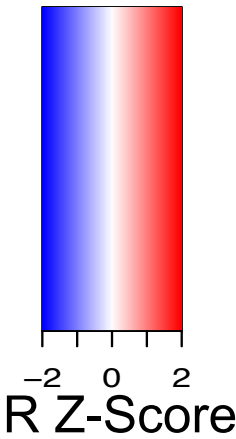

RNA-Seq Heatmap

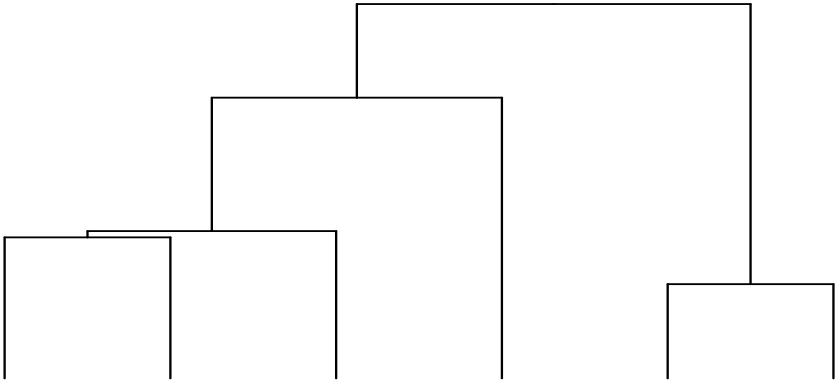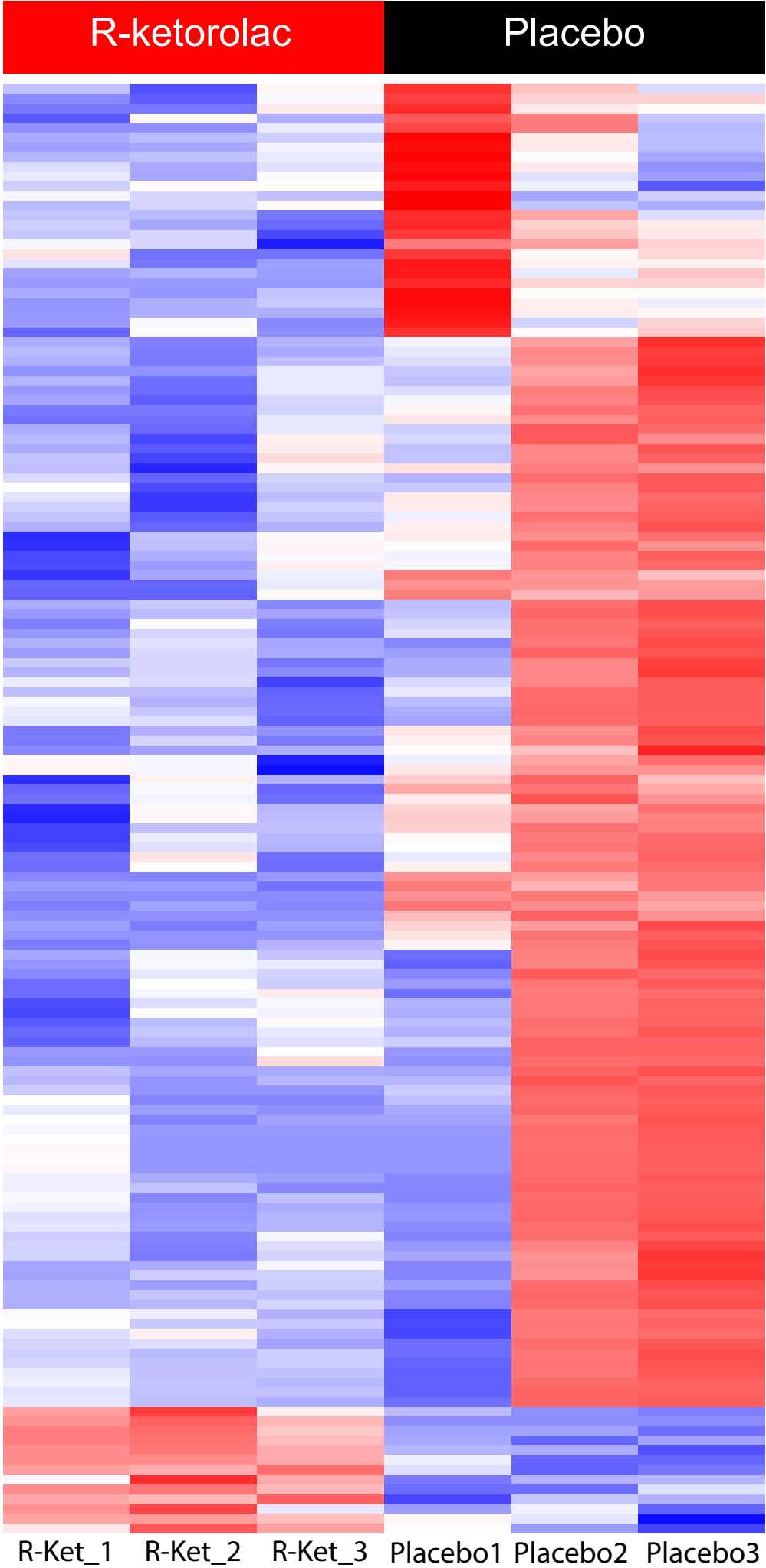

Supplement: Supplementary file 5 — Additional file 5: Figure S3. Heat map of 149 differentially expressed genes in tumors isolated from placebo versus R-ketorolac treated mice when aligned to mouse genome. Differentially expressed genes are labeled on the right and sample designations are labeled on the top. Color key in upper left indicates shading for up (red) and down (blue) regulated genes. Dendrograms at the top and side indicate relationship between the samples and differentially expressed genes, respectively. The color bar at the top indicates the sample conditions, R-ketorolac (red) and Placebo (black). [file 12885_2020_7716_MOESM5_ESM.pdf]

# HIF-1 SIGNALING PATHWAY

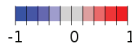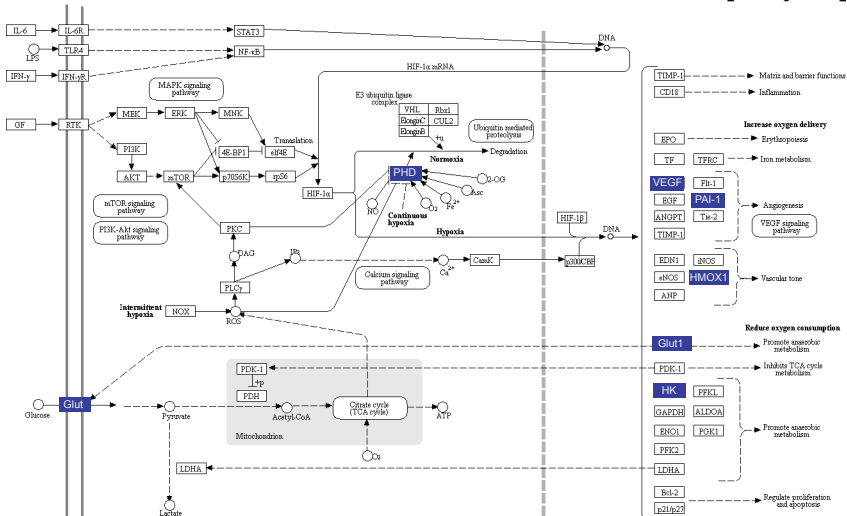

Supplement: Supplementary file 9 — Additional file 9: Figure S4. HIF-1 Signaling Pathway as defined in the KEGG Pathway database. Highlighted are the differentially expressed genes found in the tumors isolated from R-ketorolac treated mice compared to placebo control when aligned to the human genome. Color key in upper right of figure indicates shading for up (red) and down (blue) regulated genes. [file 12885_2020_7716_MOESM9_ESM.pdf]

A

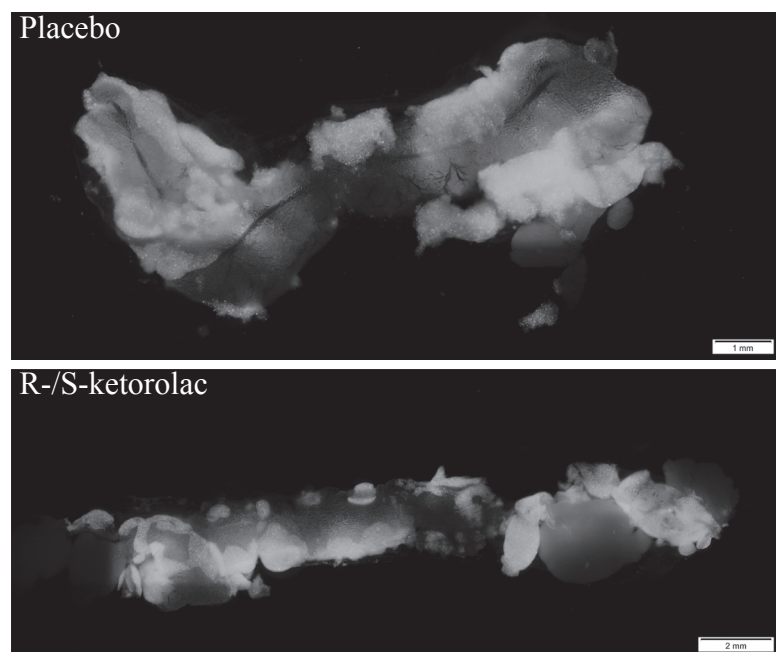

B

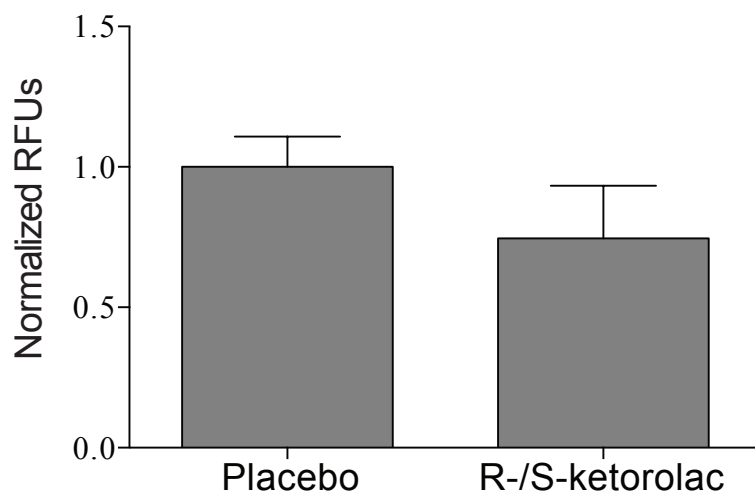

C

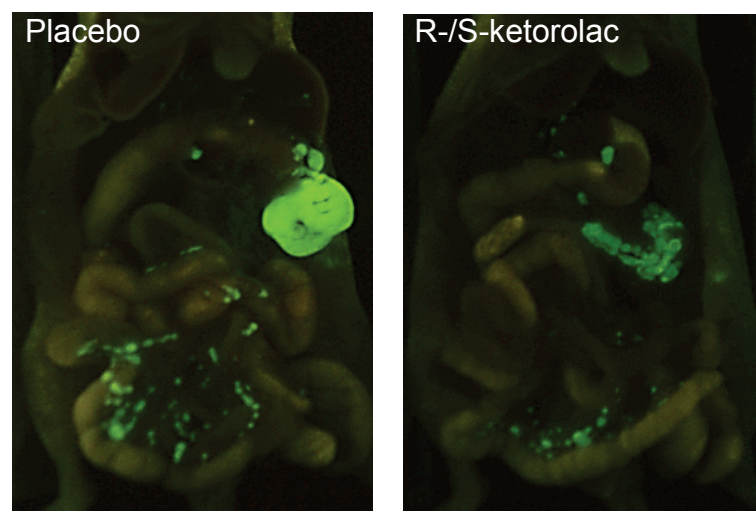

D

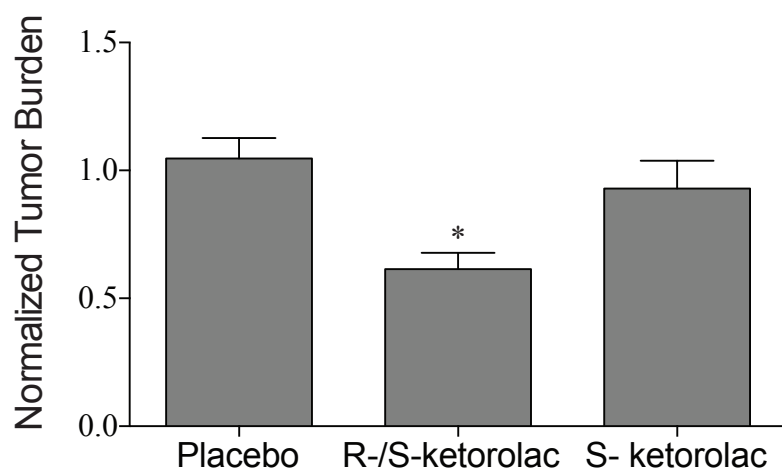

E

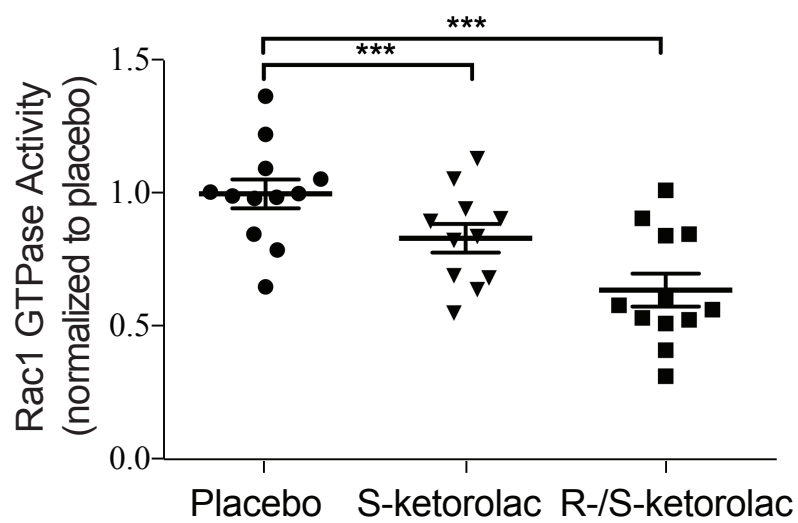

F

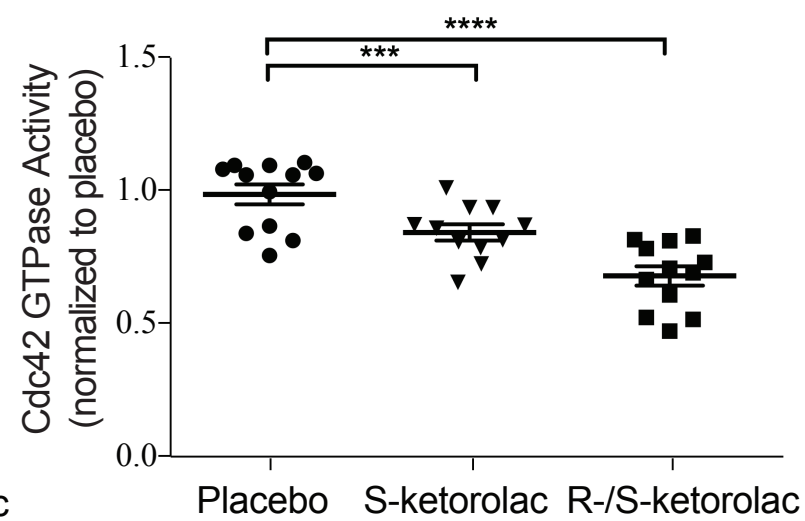

Supplement: Supplementary file 10 — Additional file 10: Figure S5. Effect of racemic and S-ketorolac on tumor burden and GTPase activity in vivo. (A) Mice were injected i.p. with SKOV3ip-GFP cells and omental engraftment was assessed after 18 h as described in Methods. Representative images of omenta isolated from animals receiving either placebo or racemic ketorolac pills. (B) Omental engraftment was quantified by GFP fluorescence and normalized to placebo treated animals within individual experiments. These data represent the combined normalized GFP fluorescence from three separate experiments with 12 total mice. Oral administration of S-ketorolac and R−/S-ketorolac reduces tumor burden in vivo. (C) Mice were injected i.p. with GFP-expressing SKOV3ip and tumors were established for 14 days. Representative images are shown of mice treated with either placebo, S-ketorolac or R−/S-ketorolac. (D) Tumor burden was quantified by counting visible tumor implants within the peritoneal cavity and normalized to placebo control mice as described in the legend to Fig. 2. Data represents three separate experiments with S-ketorolac, n = 11; R−/S-ketorolac, n = 14. * indicates p-value ≤0.05 when compared to placebo control group. GTPase activity of (E) Rac1 and (F) Cdc42 were measured in tumor lysates by a GTPase effector-binding assay as described in Methods. The data represent combined normalized activity from four separate animal experiments with GTPase activities measured in duplicate from three individual animals per experimental group, n = 12 (n = 11 for S-ketorolac). GTPase activity for Rac1: p-value ≤0.0001 for S-ketorolac and for Cdc42 p-value ≤0.001 for R−/S-ketorolac; Cdc42 GTPase activity: p-value ≤0.001 for S-ketorolac and p ≤ 0.0001 for R−/S-ketorolac when compared to placebo group. Statistical analyses were performed using one-way ANOVA, followed by Dunnett’s multiple comparisons test. Vertical bars represent SEM. [file 12885_2020_7716_MOESM10_ESM.pdf]

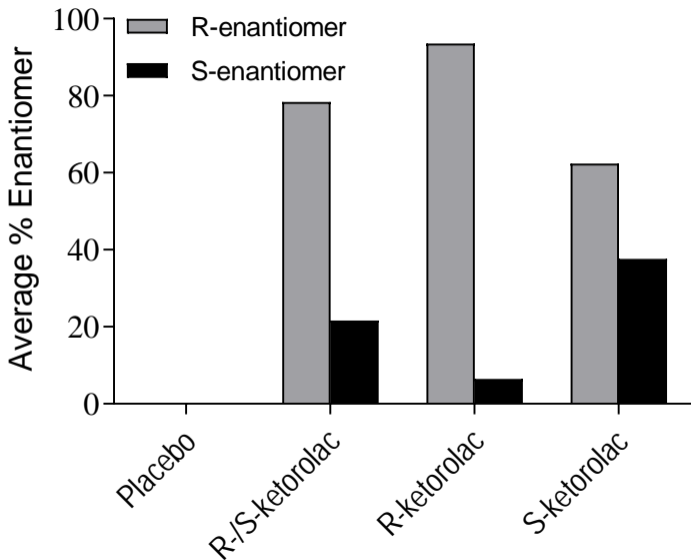

Supplement: Supplementary file 11 — Additional file 11: Figure S6. Ketorolac enantiomers in mouse serum after two-week drug treatment. Recovered ketorolac enantiomers in serum were analyzed by HPLC as described in Supplemental Methods (Additional File 1: Supplemental Methods). For each treatment group, grey bars represent average percent of R-enantiomer and black bars represent average percent of S-enantiomer. A predominance of R-ketorolac over S-ketorolac in all ketorolac treatment groups indicates an inter-conversion of S-ketorolac to R-ketorolac that occurs in mice as reported previously in the literature [28]. R-ketorolac represented approximately 88, 95, and 75% of the total recovered ketorolac from R−/S-ketorolac, R-ketorolac, and S-ketorolac treated animals, respectively after chronic administration for two weeks. Data represented are from three combined animal studies with a total of 12 mice. [file 12885_2020_7716_MOESM11_ESM.pdf]

A

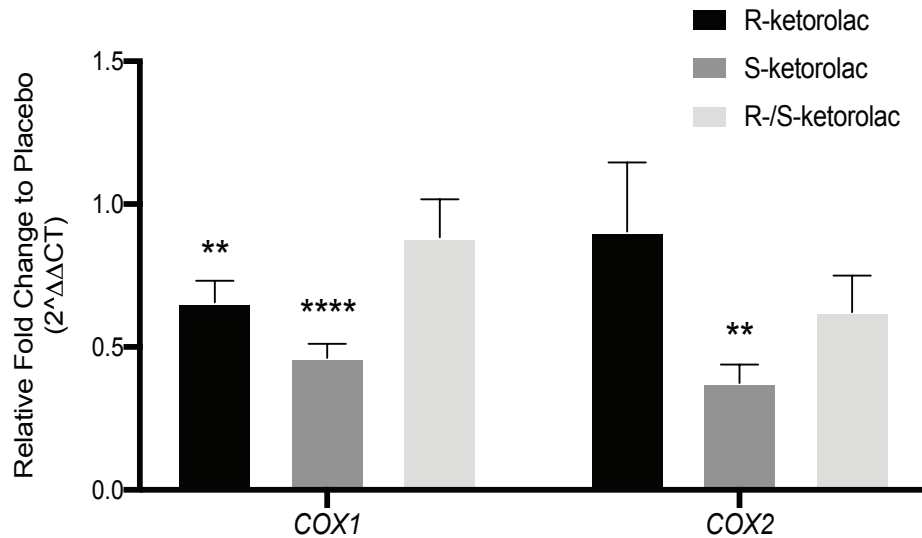

B

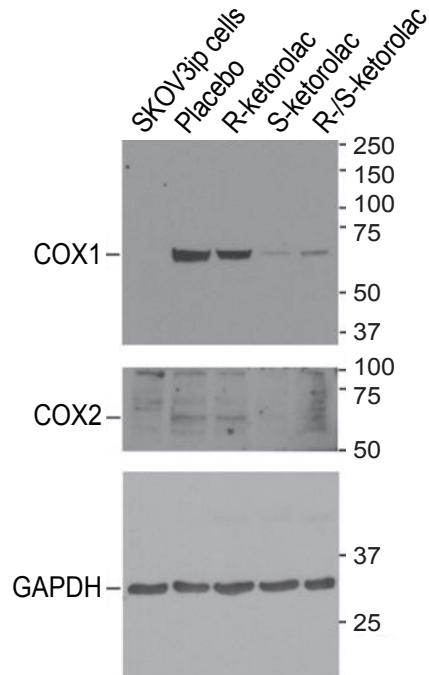

Supplement: Supplementary file 12 — Additional file 12: Figure S7. Expression of COX1 and COX2 in tumors from placebo, R-ketorolac, S-ketorolac, and racemic ketorolac (R−/S-) treated mice (all 1 mg/kg twice daily). (A) Gene expression levels of COX1 and COX2 were measured by qPCR as described in Methods. These data are combined from three separate experiments, n = 12. ** indicates p-value ≤0.01 and **** indicates p-value ≤0.0001 when compared to tumors of placebo mice and normalized to 18 s rRNA using one-way ANOVA, followed by Dunnett’s multiple comparisons test. (B) COX1 and COX2 protein levels were decreased with S-ketorolac or R−/S-ketorolac treatment, yet unaffected by R-ketorolac treatment. Tumor protein was isolated from placebo, R-ketorolac, S-ketorolac, or R−/S-ketorolac treated mice and analyzed by western blot analysis as described in Supplemental Methods (Additional File 1: Supplemental Methods). Representative blots from one of two independent experiments are shown. GAPDH served as the loading control. [file 12885_2020_7716_MOESM12_ESM.pdf]

Rac1

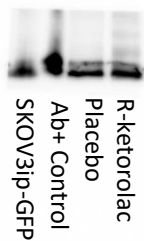

Cdc42

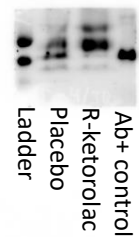

RhoA

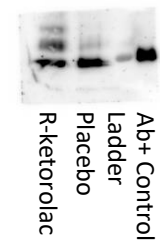

GAPDH of  
Rac1 Blot

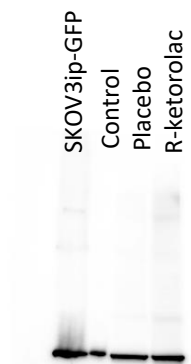

GAPDH of Cdc42 Blot

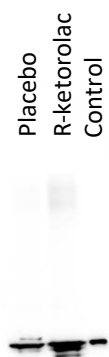

GAPDH of RhoA Blot

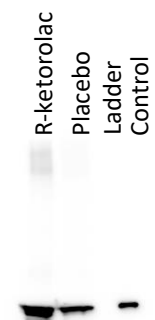

Supplement: Supplementary file 13 — Additional file 13: Figure S8. Raw images of western blot membranes used to detect Rho-GTPases in tumors from placebo and R-ketorolac treated mice. Membranes were cut between 25 and 37 kDa as guided using Precision Plus Protein Dual Color Standards (Bio-Rad Laboratories, Inc., cat# 1610374) before incubation with antibodies. This allowed probing of GAPDH (loading control) and the GTPase targets without a strip and re-probe procedure. [file 12885_2020_7716_MOESM13_ESM.pdf]

A

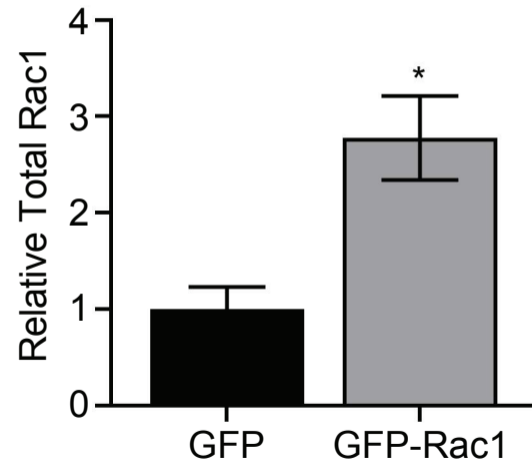

B

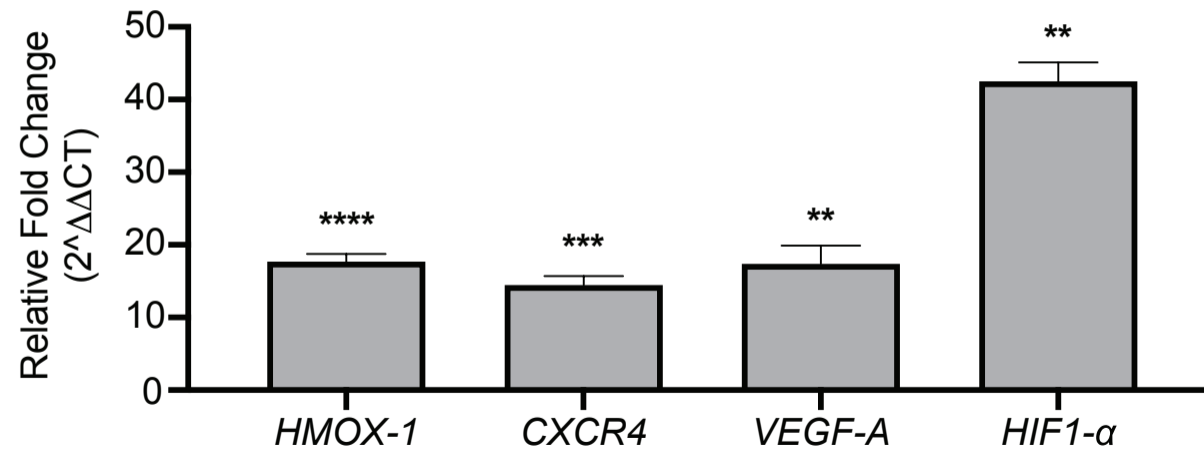

C

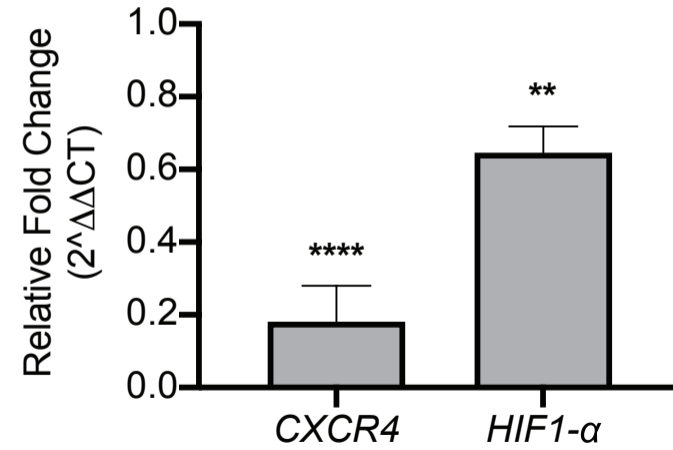

Supplement: Supplementary file 14 — Additional file 14: Figure S9. Expression of select genes showing reciprocal regulation between Rac1 over-expressing cells (SKOV3ip-GFP-Rac1) and tumors from R-ketorolac treated mice. (A) Western blot verification of Rac1 over-expressed protein of SKOV3ip-GFP-Rac1 cells relative to SKOV3ip-GFP only cells. Total Rac1 protein was normalized to GAPDH loading control. This data is from two separate experiments and was compared using unpaired t-test. (B) Gene expression levels in SKOV3ip-GFP-Rac1 cells of HMOX-1, CXCR4, VEGF-A and HIF1-α were measured by qPCR as described in Methods. These data are combined from three separate experiments. They were compared to SKOV3ip-GFP and normalized to 18 s rRNA using unpaired two tailed t-test. (C) Gene expression levels of CXCR4 and HIF1-α from tumors obtained from placebo control and R-ketorolac treated mice were determined by qPCR as described in Methods. Values are combined from three separate experiments and represent relative expression for R-ketorolac compared to placebo (1.0). Values were also normalized to 18 s rRNA and analyzed using unpaired two tailed t-test. For all panels * indicates p-value ≤0.05 ** indicates p-value ≤0.01 and **** indicates p-value ≤0.0001. [file 12885_2020_7716_MOESM14_ESM.pdf]
