# Supplementary material for: The R-enantiomer of ketorolac reduces ovarian cancer tumor burden in vivo
Source: BMC Cancer. 2021 Jan 7;21:40. doi: 10.1186/s12885-020-07716-1 (PMC7791840; doi:10.1186/s12885-020-07716-1)
Supplement: Supplementary file 3 — Additional file 3: Table S1. Omental Weight. [file 12885_2020_7716_MOESM3_ESM.pdf]

Table S1: Omental Weight

Treatment	Mean (g) ± SD	Range
Placebo	0.132 ± 0.048	0.078 - 0.17
R-ketorolac	0.059 ± 0.018	0.04 - 0.076
R-/S-ketorolac	0.058 ± 0.026	0.03 - 0.082

No significant differences between omental weights were detected between treatment groups.
