# Supplementary material for: The R-enantiomer of ketorolac reduces ovarian cancer tumor burden in vivo
Source: BMC Cancer. 2021 Jan 7;21:40. doi: 10.1186/s12885-020-07716-1 (PMC7791840; doi:10.1186/s12885-020-07716-1)
Supplement: Supplementary file 6 — Additional file 6: Table S2. topGO Categories against the Human Genome. [file 12885_2020_7716_MOESM6_ESM.pdf]

Table S2: topGO Categories against Human Genome

Rank	Category	# genes	Genes
1	Regulation of metabolic process	16	*RPE65, DEPP1, CELA1, ANGPTL4, MYADM, FOSL2, HK2, CXCR4, IGFBP5, WFDC13, SPINT4, HMOX1, DUSP1, VEGFA, SERPINE1, CA9*
2	Cell death	10	*ADM, ANGPTL4, FOSL2, HK2, CXCR4, HMOX1, DUSP1, VEGFA, SERPINE1, NDRG1*
3	Angiogenesis	9	*ADM, CELA1, ANGPTL4, HK2, NRP2, HMOX1, COL23A1, VEGFA, SERPINE1*
4	Response to hypoxia	8	*ADM, ANGPTL4, HK2, CXCR4, HMOX1, VEGFA, NDRG1, CA9*
5	Positive regulation of angiogenesis	7	*ADM, CELA1, ANGPTL4, HK2, HMOX1, VEGFA, SERPINE1*
6	Negative Regulation of Endopeptidase Activity	4	*WFDC13, SPINT4, VEGFA, SERPINE1*
7	Negative Regulation of Cell Migration	4	*IGFBP5, HMOX1, DUSP1, SERPINE1*
8	Negative Reg of Epithelial Cell Apoptotic process	3	*ANGPTL4, HMOX1, SERPINE1*
9	Positive Reg of Cell Migration in Sprouting Angiogenesis	2	*HMOX1, VEGFA*
10	Regulation of Monocyte Chemotaxis	2	*DUSP1, SERPINE1*
11	Regulation of Protein Kinase C Signaling	2	*MYADM, VEGFA*
12	Myelin Maintenance	2	*CXCR4, NDRG1*
13	Pos Reg of Blood Vessel Endothelial Cell Prolif in Angio	2	*HMOX1, VEGFA*
14	Vascular Smooth Muscle Cell Development	2	*ADM, VEGFA*
