# Supplementary material for: The R-enantiomer of ketorolac reduces ovarian cancer tumor burden in vivo
Source: BMC Cancer. 2021 Jan 7;21:40. doi: 10.1186/s12885-020-07716-1 (PMC7791840; doi:10.1186/s12885-020-07716-1)
Supplement: Supplementary file 7 — Additional file 7: Table S3. topGO Categories against the Mouse Genome. [file 12885_2020_7716_MOESM7_ESM.pdf]

Table S3: topGO Categories against Mouse Genome

Rank	Category	# genes	Genes
1	Proteolysis	29	*Serpinb2, Ctse, Ptgs2, Ggt1, Usp43, Vtn, Ero1lb, Itih4, Cma1, Cela1, Tpsab1, Vegfa, Spint1, Slpi, Cpb1, Egf, Cela3b, Cela2a, Ctrc, Hgfac, Cpa2, Cpa1, 2210010C04Rik, Try5, Klk1, Cuzd1, Brsk2, Ctrl, Arxes2*
2	Response to Bacterium	14	*Ptgs2, Slamf8, Ggt1, Nos2, Tlr11, Clps, Clps, Pnliprp2, Lbp, Slpi, Amy1, Clec4d, Dmbt1, Nod2, Mt2*
3	Cellular Metal ion Homeostasis	12	*Pkhd1, Slc41a1, Slc39a5, Gck, Stc2, Ctrc, Cckar, Trpv6, Gipr, Nucb2, Mt2, Mt1*
4	Cellular Divalent Inorganic Cation Homeostasis	12	*Pkhd1, Slc41a1, Slc39a5, Gck, Stc, Ctrc, Cckar, Trpv6, Gipr, Nucb2, Mt2, Mt1*
5	Response to Starvation	9	*Slc39a5, Gck, 5330417C22Rik, Rragd, Slc2a1, Cckar, Bhlha15, Asns,*
6	Insulin Secretion	8	*Gck, Nos2, Ptprn2, Pclo, Cckar, Hnf1a, Gipr, Brsk2*
7	Regulation of Peptide Hormone Secretion	7	*Gck, Nos2, Cckar, Hnf1a, Gipr, Nucb2, Brsk2*
8	Negative Regulation of Peptidase Activity	7	*Serpinb2, Ptgs2, Vtn, Itih4, Vegfa, Spint1, Slpi*
9	Positive Regulation of Endocytosis	6	*Vtn, Vegfa, Lbp, Egf, Rap1gap, Nod2*
10	Regulation of Glucose Metabolic Process	5	*Gck, Gnmt, Pdk1, Lcmt1, Ppp1r3b*
11	Alpha-Amino Acid Biosynthetic Process	5	*Ggt1, Gls2, Cbs, Asns, Aass*
12	Aspartate Family Amino Acid Metabolic Process	5	*Mat1a, Gcat, Gnmt, Asns, Aass*
13	Nitric Oxide Mediated Signal Transduction	5	*Nos2, Cbs, Vegfa, Mt2, Mt1*
14	Protein Homotetramerization	5	*Pcbd1, Mat1a, Gnmt, Trpv6, Reg1*
15	SMAD Protein Signal Transduction	4	*Inhba, Gata4, Bmp3, Hnf1a*
16	One-Carbon Metabolic Process	4	*Aldh1l2, Mat1a, Gnmt, Mthfd2*
17	Pteridine-Containing Compound Metabolic Process	4	*Pcbd1, Aldh1l2, Ggh, Mthfd2*
18	Glutamine Family Amino Acid Metabolic Process	4	*Ggt1, Gls2, Nos2, Asns*
19	Endoderm Formation	3	*Vtn, Inhba, Gata4*
20	Response to Zinc Ion	3	*Ggh, Mt2, Mt1*
21	Stress Response to Metal Ion	2	*Mt2, Mt1*
22	Cysteine Biosynthetic Process	2	*Ggt1, Cbs*
23	Respiratory Burst Involved in Inflammatory Response	2	*Slamf8, Lbp*
24	Cellular Response to Follicle-Stimulating Hormone Stimulus	2	*Inhba, Gata4*
25	S-adenosylmethionine Metabolic Process	2	*Mat1a, Gnmt*
26	Insulin Metabolic Process	2	*Ero1lb, Nucb2*
27	Response to Magnesium Ion	2	*Slc41a1, Ank3*
28	Maintenance of Gastrointestinal Epithelium	2	*Tff2, Nod2*
