# Supplementary material for: The R-enantiomer of ketorolac reduces ovarian cancer tumor burden in vivo
Source: BMC Cancer. 2021 Jan 7;21:40. doi: 10.1186/s12885-020-07716-1 (PMC7791840; doi:10.1186/s12885-020-07716-1)
Supplement: Supplementary file 8 — Additional file 8: Table S4. Significant KEGG Pathways using the Mouse Genome. [file 12885_2020_7716_MOESM8_ESM.pdf]

Table S4: Significant KEGG Pathways using Mouse Genome

Rank	Category	# genes	p-value	Genes
1	Pancreatic secretion	12	4.74E-10	*Rab27b, Pnlipro2, Cpb1, Amy1, Cela3b, Cela2a, Cckar, Cpa2, Cpa1,*
2	Protein digestion and absorption	8	3.48E-06	*Cpb1, Cela3b, Cela2a, Cpa2, Cpa1, 2210010C04Rik, Try5, Ctrl*
3	Maturity onset diabetes of the young	3	2.26E-03	*Gck, Hnf1a, Bhlha15*
4	Central carbon metabolism in cancer	4	4.16E-03	*Gls2, Gck, Pdk1, Slc2a1*
5	HIF-1 signaling pathway	5	5.17E-03	*Nos2, Vegfa, Pdk1, Egf, Slc2a1*
6	Glycine serine and threonine metabolism	3	6.96E-03	*Gcat, Cbs, Gnmt*
7	Insulin secretion	4	1.00E-02	*Gck, Slc2a1, Pclo, Cckar*
8	Mineral absorption	3	1.09E-02	*Mt2, Mt1*
9	GABAergic synapse	4	1.17E-02	*Gls2, Gabra4, Slc38a3, Slc38a5*
10	Arginine biosynthesis	2	1.46E-02	*Gls2, Nos2*
11	One carbon pool by folate	2	1.46E-02	*Aldh1l2, Mthfd2*
12	Proximal tubule bicarbonate reclamation	2	1.94E-02	*Gls2, Slc38a3*
13	Folate biosynthesis	2	2.66E-02	*Pcbd1, Ggh*
14	D-Glutamine and D-glutamate metabolism	1	3.87E-02	*Gls2*
15	Biosynthesis of amino acids	3	4.11E-02	*Mat1a, Cbs, Asns*
16	Starch and sucrose metabolism	2	4.14E-02	*Gck, Amy1*
17	Focal adhesion	5	4.58E-02	*Vtn, Vegfa, Lamc3, Egf, Spp1*
18	Neomycin kanamycin and gentamicin biosynthesis	1	4.82E-02	*Gck*
19	Renin-angiotensin system	2	4.84E-02	*Cma1, Klk1*
